# PriTKT: A Blockchain-Enhanced Privacy-Preserving Electronic Ticket System for IoT Devices

**DOI:** 10.3390/s24020496

**Published:** 2024-01-13

**Authors:** Yonghua Zhan, Feng Yuan, Rui Shi, Guozhen Shi, Chen Dong

**Affiliations:** 1 College of Computer and Data Science, Fuzhou University, Fuzhou 350108, China; 190310007@fzu.edu.cn (Y.Z.); dongchen@fzu.edu.cn (C.D.); 2 Institute 706, The Second Academy China Aerospace Science & Industry Corp, Beijing 100854, China; fyuan1234@aliyun.com; 3 Beijing Electronic Science and Technology Institute, Beijing 100070, China; sgz1974@163.com

**Keywords:** electronic tickets, privacy-preserving, blockchain, IoT, double-spending detection

## Abstract

Electronic tickets (e-tickets) are gradually being adopted as a substitute for paper-based tickets to bring convenience to customers, corporations, and governments. However, their adoption faces a number of practical challenges, such as flexibility, privacy, secure storage, and inability to deploy on IoT devices such as smartphones. These concerns motivate the current research on e-ticket systems, which seeks to ensure the unforgeability and authenticity of e-tickets while simultaneously protecting user privacy. Many existing schemes cannot fully satisfy all these requirements. To improve on the current state-of-the-art solutions, this paper constructs a blockchain-enhanced privacy-preserving e-ticket system for IoT devices, dubbed PriTKT, which is based on blockchain, structure-preserving signatures (SPS), unlinkable redactable signatures (URS), and zero-knowledge proofs (ZKP). It supports flexible policy-based ticket purchasing and ensures user unlinkability. According to the data minimization and revealing principle of GDPR, PriTKT empowers users to selectively disclose subsets of (necessary) attributes to sellers as long as the disclosed attributes satisfy ticket purchasing policies. In addition, benefiting from the decentralization and immutability of blockchain, effective detection and efficient tracing of double spending of e-tickets are supported in PriTKT. Considering the impracticality of existing e-tickets schemes with burdensome ZKPs, we replace them with URS/SPS or efficient ZKP to significantly improve the efficiency of ticket issuing and make it suitable for use on smartphones.

## 1. Introduction

E-ticketing has emerged as a popular method of ticket entry, processing, and marketing for companies in the airline [1], railway [2,3], and other transportation and entertainment industries. The integration of blockchain technology in e-ticketing has further enhanced the security and transparency of transactions. Blockchain [4,5,6,7] ensures that each transaction is recorded in a decentralized and tamper-proof ledger, reducing the risk of fraud and ensuring the authenticity of e-tickets. Unlike traditional paper-based tickets, e-tickets offer two major advantages. First, paper-based tickets are disposable and require significant resources for their production, leading to an increased negative impact on the environment. By contrast, e-tickets serve as an eco-friendly replacement by minimizing paper waste produced from ticketing activities, thereby aligning with the principles of the Paris Agreement [8]. Second, e-tickets provide customers with the flexibility to reserve, issue, and refund their tickets online anytime and anywhere, eliminating the need for in-person lineups. This convenience and ease for customers is further heightened by the integration of IoT technology. Customers can easily access their e-tickets through dedicated mobile applications, making the entire ticketing process more streamlined and accessible. Seamless integration with IoT devices not only enhances the overall customer experience, it reflects the evolving nature of ticketing systems in the digital age. As a result, e-tickets, powered by blockchain and IoT technology, offer significant advantages in terms of convenience, environmental sustainability, and security.

Despite the increasing popularity of e-tickets, they face numerous practical challenges, particularly with regards to privacy. In e-ticket systems, user data such as name, identity number, purchase date, and other personal attributes may be collected and misused. Thus, it is crucial to minimize the collection of personal data in line with the recently introduced General Data Protection Regulations (GDPR) [9]. In this regard, various privacy-protecting e-ticket systems have been proposed which use randomizable signatures with efficient proofs [10,11,12,13,14], pseudonyms [15,16], and anonymous credentials [13,17,18]. However, some of these systems lack formal proof of security and cannot guarantee the integrity of the token [12,13,16], while others are inefficient in their invoicing operations, making them impossible to deploy on IoT devices [18].

An essential feature of any e-ticket system is the ability to issue tickets based on user attributes. Attribute-based e-ticket systems have potential in various real-world applications. For example, they can enable students, soldiers, and individuals with disabilities to purchase tickets at discounted rates without revealing sensitive information such as a student ID number, unit number, or health conditions. In an attribute-based e-ticketing system, a user credential used to purchase e-tickets is parameterized with a vector of the user’s attributes, such as date of birth, affiliation, or occupation. During ticket purchase, users can prove that they possess a credential meeting a given attribute policy, such as age or disability, without revealing any additional information beyond the satisfaction of the attribute policy. Unfortunately, most existing e-ticketing systems [12,13,14,16] do not support attribute-based ticket issuance protocols.

Credentials issued by governmental bodies, schools, or companies typically include only the basic attributes of the user, such as name, gender, educational background, address, and position. However, in many cases additional attributes can be defined as well. For instance, in platform configuration-based access control services, attributes may refer not only to the user’s personal data but to hardware platform and configuration information. Moreover, one user may hold several roles, each with unique credentials issued by a single entity. The Privacy by Design Foundation’s Anonymous Credentials (IRMA) [19] offers a broad range of real-world attributes, such as diplomas, passports, cards, and even membership IDs for online services, which are relevant to governing bodies and various businesses. Such scenarios imply that a user may have hundreds or more potential attributes. Integrating IoT devices into the e-ticketing system allows for the inclusion of additional attributes related to the user while presenting an opportunity to streamline the issuance process. IoT devices capabilities, such as bio-metric authentication and secure storage, can enhance the security and efficiency of handling a wide array of attributes. This ensures that the e-ticketing system remains practical and user-friendly even in scenarios with a large number of potential attributes. While Han et al. [18] designed the first attribute-based e-ticketing system, their ticket issuance algorithm’s computational cost and communication overhead increases linearly with the number of user attributes, making their scheme impractical to deploy on IoT devices when users have hundreds of attributes.

Paper-based tickets can be made unique, and are easily distinguishable from copies; however, distinguishing original e-tickets from their copied versions is challenging, making it necessary to prevent and detect double spending in e-ticketing systems. This challenge is particularly pertinent in the digital realm, where the ease of replication poses a unique set of security concerns. The integration of blockchain technology offers a promising solution to the issue of double spending in e-ticketing. By utilizing a decentralized and tamper-proof ledger, blockchain ensures the immutability and transparency of transaction records. Each e-ticket transaction can be securely recorded on the blockchain, creating a verifiable and unforgeable trail. This prevents unauthorized duplication of e-tickets while allowing for efficient tracking and identification of any double-spending attempts. Moreover, in the context of IoT technology, blockchain can be seamlessly integrated to enhance the security of e-tickets. IoT devices can serve as secure digital wallets, storing and managing e-tickets in a tamper-resistant environment. The combination of blockchain and IoT devices ensures that the integrity of e-tickets is maintained, reducing the risk of duplication and unauthorized use. In the event of a double-spending attempt, the decentralized nature of blockchain enables the identification and tracing of responsible users without compromising the anonymity of honest users. The transparency of the blockchain allows for swift and accurate resolution of security incidents, thereby bolstering the overall trust and reliability of the e-ticketing system.

Based on the above requirements and intuition, we propose PriTKT, a blockchain-enhanced privacy-preserving e-ticketing system for IoT devices that supports ticket issuance based on user attributes. PriTKT significantly reduces the computational cost and communication overhead, making it suitable for use on IoT devices, and utilizes blockchain [4] to achieve effective malicious user tracking.

### Our Contributions

This work makes the following contributions:

•**Attribute-based ticketing**: We propose an e-ticket system that supports attribute-based ticketing. This system securely and seamlessly integrates attribute-based anonymous credentials [20], unlinkable redactable signatures (URS) [20], structure-preserving signatures (SPS) [21], Pedersen commitment, and zero-knowledge signature of knowledge (ZKSoK) [22] in its design. In our e-ticket system, a trusted party generates a ticket purchasing policy set during system initialization. The ticket purchasing policy set regulates how tickets are issued. User privacy is preserved, as users may disclose a subset of their attributes as long as they satisfy a ticket purchasing policy in the ticket purchasing policy set for the tickets they purchase.

•**Efficient ticketing for IoT devices**: Our system improves the efficiency of the ticketing algorithm to ensure that it can be efficiently executed on an IoT device (such as smartphone) while presenting a privacy-preserving e-ticketing solution that does not require expensive Zero-Knowledge Proofs (ZKP) to validate user credentials during ticket purchase. Instead, we use the URS signature, which reduces the computational overhead in the ticket-issuing algorithm from O(N) to O(N−K) and the communication overhead from O(N) to O(K) , where *N* and *K* represent the number of user attributes and exposed attributes, respectively. Compared to the closest scheme in [18], our method improves both computational and communication performance. We implemented PriTKT on a smartphone under AES-100-bit security and compared it with the state-of-the-art scheme from [18], finding that our issuing algorithm and showing algorithm were 250% and 240% more efficient, respectively.

•**Blockchain-enhanced double-spending detection and efficient trace**: We implement double-spending detection and efficient user tracking. The verifier uploads each valid token into the blockchain. The decentralization and immutability of the blockchain [4] ensures the correctness of the token storage. Every ticket must disclose its unique identity when it is shown, and ZKSoK is used to guarantee the correctness of its identity disclosure. With the ticket identity, the verifier can traverse the tokens stored in the blockchain and quickly detect any double-spending. Using the “Schnorr trick” [23], the verifier can quickly compute the public key of any double-spending user for efficient tracing.

•**Unlinkability and framing resistance**: Our solution ensures the unlinkability of tickets. Using the unlinkability of URS signatures, we prove that no tickets of the same user can be linked. Our solution ensures the framing resistance of tickets. Framing resistance ensures that corrupt sellers cannot falsely accuse any honest users of double-spending.

In addition, we offer formal security definitions for e-tickets that can be reduced to known complexity assumptions or the security of established cryptography primitives. The performance of PriTKT was measured on a smartphone.

## 2. Related Work

### 2.1. Electronic Tickets on IoT Devices

Mut-Puigserver et al. [24] conducted a study on the functional and security requirements of electronic tickets. These requirements include offline verification, expiration dates, reduced size, portability, flexibility, unlinkability, unforgeability, and non-overspending. They explored various types of e-tickets, such as single-use, multi-use, transferable, and non-transferable. Our research specifically focuses on analyzing the unlinkability, unforgeability, and non-overspending features of single-use and non-transferable electronic tickets leveraging the capabilities of IoT devices and the security of blockchain technology.

Previous studies by Heydt-Benjamin et al. [12] employed electronic cash, anonymous credentials [25], and proxy re-encryption [26] to enhance privacy in public transit systems that use electronic tickets. The study proposed a theoretical framework to examine the security of payments and the privacy of information in transit systems. They asserted that their system was capable of safeguarding user privacy but was not compatible with attribute-based ticketing; however, the paper did not provide any formal proof to support the system’s security. In contrast, the integration of IoT technology and blockchain in modern e-ticketing systems, such as PriTKT, can offer improved security measures, including attribute-based ticketing, while maintaining user privacy. Following Rupp et al. [14], Jager et al. [27] presented Black-Box Accumulation (BBA) to establish cryptography payment systems. Later, BBA was enhanced to BBA+ [28] by Hartung et al. and Black-Box Wallet (BBW) by Hoffmann et al. [29]. Nonetheless, these payment schemes [27,28,29] differ from attribute-based e-ticketing systems, as they pertain to electronic payments.

Vives-Guasch et al. [16] introduced an e-ticketing system that considers user privacy requirements as well as security requirements that include exculpability and re-usability. Additionally, by utilizing lightweight cryptography and mobile phones equipped with Near-Field Communication (NFC) technology, their system accommodates the computational limitations of users. Regrettably, however, their system does not support attribute-based ticketing. The integration of IoT devices (smartphones) in e-ticketing systems, as demonstrated by PriTKT, can contribute to enhanced features such as de-anonymization prevention and secure ticket non-transferability, while blockchain ensures the security and transparency of transactions.

The system of Milutinovic et al. [13] depends on certified tokens that are impossible to relate, and on different cryptographic primitives such as commitment schemes [30], partially blind signatures [31], and anonymous credentials [32] to tackle privacy concerns. However, their system does not provide de-anonymization capabilities after double spending or support ticket non-transferability in the way that PriTKT does. The integration of IoT devices capabilities in PriTKT enhances security measures and provides a practical solution for these challenges.

Han et al. [18] introduced attribute-based credentials derived from the Boneh–Boyen signature (BBS) [33] and efficient set membership proof and range proofs [34] to issue attribute credentials and tickets. However, due to the use of a signature with the NIZK protocol, their system’s NIZK proof computation increases linearly with the number of attributes in the ticket-issuing algorithm, limiting its practical use. The integration of lightweight and efficient IoT technology, along with the security of blockchains, can potentially address these computational constraints, making attribute-based e-ticketing more practical and user-friendly.

### 2.2. Blockchain-Enhanced Double-Spending Detection

Double-spending problems exist not only in e-ticket system [12,13,14,16,18], but in electronic payment systems [23,27,28,29] and blockchain cryptocurrency schemes [5,6,7]. Blockchain technology plays a crucial role in mitigating the double-spending issue by providing a decentralized and transparent ledger that ensures the integrity and uniqueness of transactions.

The most direct way to prevent double-spending is to bind each spending operation with a unique identifier that cannot be forged to ensure that that verifiers can detect double-spending based on the unique identifier. Rupp et al. [14] used a digital signature as the identifier. Sasson et al. [5] utilized hash-based commitments to compute identifiers. Androulaki et al. [6] utilized verifiable random functions to generate identifiers. Han et al. [18] and Sun et al. [7] used elements randomly mapped to elliptic curve groups as identifiers. Jager et al. [27] and Bobolz et al. [23] directly used a random number as the identifier. Although there are many ways to generate identifiers, these schemes all need to compute a zero-knowledge proof in the spending protocol to prove the correctness of the disclosed identifier.

Certain schemes [5,6,7,12,13,16,27] simply terminate the spending protocol when a double-spend is detected, while others [14,18,23,28,29] support further tracing and identification of malicious users. The method used by Han et al. [18] was to hide users’ identity in ElGamal ciphertext, then use a verifier to recover their identities through the spending algorithms. The scheme in [14,23,28,29] achieved double-spending tracing through the “Schnorr trick”, which is more efficient than scheme in [18]. The integration of blockchains into these schemes can enhance the overall security and transparency of their double-spending prevention and tracing mechanisms.

### 2.3. Attribute-Based Credentials

Anonymized attribute-based credentials that enable selective disclosure of attributes can be obtained in a manner akin to the use of randomizable signatures. Every user receives a signature on (commitments to) a list of attributes from a centralized authority. When the credential is presented, the user randomizes the signature (ensuring that the resulting signature and the published signature cannot be linked) and proves the correspondence of this signature to the revealed and hidden attributes in zero-knowledge proofs [35,36,37,38,39,40,41]. From a privacy perspective, this solution is perfectly satisfactory; however, it is not very efficient, as the user’s unshared attributes impose more cost than the user’s revealed attributes.

Fuchsbauer and Hanser [42,43] randomized both the signature and the signed message (which is a set commitment to the user’s attributes) with a structure-preserving signature on equivalence classes (SPS-EQ), then used subset opening of the set commitments to selectively disclose attributes. In this way, they avoided the need to perform costly ZKP over the hidden attributes. Unfortunately, while attributes can be disclosed in this solution once they are signed, it cannot be proven that they are hidden while satisfying certain relations.

Camenisch et al. [44] presented a novel unlinkable redactable signature (URS) that allows part of the signed message to be redacted while proving that the signature is valid for the disclosed attributes. Unfortunately, their scheme can only be instantiated by Groth–Sahai proofs [45], and it is difficult to compete with the most effective solution in practice. Sanders followed the URS approach from [44] and constructed a flexible redactable signature scheme that achieves unlinkability at almost zero cost. Unlike the methods in [43,44], the URS presented by Sanders can prove complex relationships between attributes and does not rely on zero-knowledge proofs for partial verification.

## 3. Preliminaries

### 3.1. Bilinear Pairing

Suppose that $G_{1}$ , $G_{2}$ , and $G_{T}$ are groups of prime-order *p* with generators $g\in G_{1}$ and $\tilde{g}\in G_{2}$ . A mapping $e:G_{1}\times G_{2}\to G_{T}$ is a bilinear map if it satisfies three requisite properties. (1) *Bilinearity*: for all $g\in G_{1}$ , $\tilde{g}\in G_{2}$ , and $a,b\in Z_{p}$ , we have $e\left(g^{a},{\tilde{g}}^{b}\right)=e{\left(g,\tilde{g}\right)}^{ab}$ ; (2) *Nondegeneracy*: $e(g,\tilde{g})\neq 1$ ; and (3) *Computability*: *e* is an efficiently computable function. The PriTKT scheme is based on the Type III bilinear pairing [46], which by definition does not admit an efficiently computable homomorphism between $G_{1}$ and $G_{2}$ .

### 3.2. Computational Assumptions

Discrete Logarithm (DL) Assumption. Let G be a prime-order cyclic group and let *g* be a generator of G . Given $\left(g,g^{x}\right)\in G^{2}$ , the DL assumption holds in G if no efficient algorithm or adversary can practically compute *x* with non-negligible probability.

The DDH Assumption. Let G be a prime-order cyclic group and let *g* be a generator of G . Given $\left(g,g^{x},g^{y},g^{z}\right)\in G^{4}$ , the DDH assumption holds in *G* if no efficient adversary can distinguish the product z=x·y from a random element in G .

### 3.3. Unlinkable Redactable Signatures

The Unlinkable Redactable Signature (URS) [20] consists of a tuple (Setup, KeyGen, Sign, Derive, Verify) of probabilistic polynomial-time (PPT) algorithms. The URS is used to issue credentials for a user’s attributes.

$•\;\mathrm{Setup}(1^{\lambda })$ : given a security parameter λ , this algorithm generates the public parameters $pp=(G_{1},G_{2},G_{T},g,\tilde{g},p,e)$ .

•KeyGen(n) : given an input integer *n*, this algorithm selects $\left(x,y_{1},\ldots ,y_{n}\right)\overset{\;R\;}{\leftarrow }Z_{p}^{n+1}$ and computes $X=g^{x}$ , $Y_{i}=g^{y_{i}}$ , ${\tilde{Y}}_{i}={\tilde{g}}^{y_{i}}$ , 1⩽i⩽n , and $Z_{i,j}=g^{y_{i}\cdot y_{j}}$ , 1⩽i≠j⩽n . Then, the secret key is $sk=(x,y_{1},\ldots ,y_{n})$ and the public key is $pk=(X,{\left\{Y_{i},{\tilde{Y}}_{i}\right\}}_{i=1}^{n},{\left\{Z_{i,j}\right\}}_{1⩽i\neq j⩽n})$ .

$•\;\mathrm{Sign}(sk,A={\left\{a_{i}\right\}}_{i=1}^{n})$ : to sign *n* messages $a_{1},\ldots ,a_{n}$ ( $a_{i}\in Z_{p},1⩽i⩽n$ ), this algorithm selects ${\tilde{\sigma }}_{1}\overset{\;R\;}{\leftarrow }G_{2}$ and computes ${\tilde{\sigma }}_{2}=\sigma_{1}^{x+\sum_{i=1}^{n}y_{i}\cdot a_{i}}$ . It sets $\sigma_{1}=1_{G_{1}}$ and $\sigma_{2}=1_{G_{1}}$ , then outputs $\sigma =(\sigma_{1},\sigma_{2},{\tilde{\sigma }}_{1},{\tilde{\sigma }}_{2})$ .

•Derive(pk,σ,A,D) : given an input signature $\sigma =(\sigma_{1},\sigma_{2},$ ${\tilde{\sigma }}_{1},{\tilde{\sigma }}_{2})$ on ${\left\{a_{i}\right\}}_{i=1}^{n}$ , the public key pk , and a subset D⊆[n] , this algorithm selects $\left(r,t\right)\overset{\;R\;}{\leftarrow }Z_{p}^{2}$ and computes ${\tilde{\sigma }}_{1}^{\prime }={\tilde{\sigma }}_{1}^{r}$ , ${\tilde{\sigma }}_{2}^{\prime }={\tilde{\sigma }}_{2}^{r}{\left({\tilde{\sigma }}_{1}^{\prime }\right)}^{t}$ , $\sigma_{1}^{\prime }=g^{t}\prod_{i\in [n]\setminus D}Y_{i}^{a_{i}}$ , and $\sigma_{2}^{\prime }={\left(\prod_{i\in D}Y_{i}\right)}^{t}\prod_{i\in D,j\in [n]\setminus D}Z_{i,j}^{a_{j}}$ . If D=[n] , then [n]∖D=∅ and $\sigma_{1}^{\prime }=g^{t}$ and $\sigma_{2}^{\prime }={\left(\prod_{i\in [n]}Y_{i}\right)}^{t}$ . The algorithm returns the derived signature $\sigma^{\prime }=\left(\sigma_{1}^{\prime },\sigma_{2}^{\prime },{\tilde{\sigma }}_{1}^{\prime },{\tilde{\sigma }}_{2}^{\prime }\right)$ on D⊆[n] .

$•\;\mathrm{Verify}(pk,\sigma ,D={\left\{a_{i}\right\}}_{i\in D})$ : a signature $\left(\sigma_{1},\sigma_{2},{\tilde{\sigma }}_{1},{\tilde{\sigma }}_{2}\right)$ on D⊆[n] is valid if the following equations hold: $e(X\sigma_{1}\cdot \prod_{i\in D}{\tilde{Y}}_{i}^{a_{i}},$ ${\tilde{\sigma }}_{1})=e\left(g,{\tilde{g}}_{2}\right)$ and $e\left(\sigma_{1},\prod_{i\in D}{\tilde{Y}}_{i}\right)=e\left(\sigma_{2},\tilde{g}\right)$ , in which case the algorithm returns 1; otherwise, it returns 0.

The URS [20] is a redactable signature scheme with forgeability and unlinkability in the generic group model.

### 3.4. Structure-Preserving Signatures

The Structure-Preserving Signatures (SPS) proposed by Groth [21] consist of a tuple (Setup,KeyGen,Sign,Verify) of PPT algorithms that sets messages in $G_{2}$ . This scheme is used to issue credentials for a seller’s public key.

$•\;\mathrm{Setup}(1^{\lambda })$ : given a security parameter $1^{\lambda }$ , this algorithm generates public parameters $pp=(G_{1},G_{2},G_{T},g,\tilde{g},p,e)$ .

•KeyGen(n) : this algorithm generates a secret key $sk=(x,y_{1},\ldots ,y_{n})$ and a public key $pk=(h;\tilde{X};{\left\{{\tilde{Y}}_{i}\right\}}_{i=1}^{n-1})$ when given an input integer *n*. It selects $\left(x,y_{1},\ldots ,y_{n-1}\right)\overset{\;R\;}{\leftarrow }Z_{p}^{n}$ and a random generator $h\overset{\;R\;}{\leftarrow }G_{1}$ , and computes $\tilde{X}={\tilde{g}}^{x}$ , ${\tilde{Y}}_{i}={\tilde{g}}^{y_{i}}$ , and 1⩽i⩽n−1 .

$•\;\mathrm{Sign}(sk,{\left\{m_{i}\right\}}_{i=1}^{n})$ : to sign *n* messages $\left(m_{1},\ldots ,m_{n}\right)$ , where $m_{i}$ is an element of group $G_{1}$ for 1≤i≤n , the signer randomly selects $r\overset{\;R\;}{\leftarrow }Z_{p}$ and computes ${\tilde{\delta }}_{1}={\tilde{g}}^{r^{-1}}$ , $\delta_{2}={\left(h\cdot g^{x}\right)}^{r}$ , and $\delta_{3}={\left(h\cdot m_{n}\cdot \prod_{i=1}^{n-1}m_{i}^{y_{i}}\right)}^{r}$ . Next, the signer outputs the signature $\delta =({\tilde{\delta }}_{1},\delta_{2},\delta_{3})$ .

$•\;\mathrm{Verify}(pk,\delta ,{\left\{m_{i}\right\}}_{i=1}^{n})$ : for verification of the signature $\delta =({\tilde{\delta }}_{1},\delta_{2},\delta_{3})$ associated with messages ${\left\{m_{i}\right\}}_{i=1}^{n}$ , two pairing equations need to be evaluated: $e\left(\delta_{2},{\tilde{\delta }}_{1}\right)=e\left(h,\tilde{g}\right)e\left(g,\tilde{X}\right)$ and $e\left(\delta_{3},{\tilde{\delta }}_{1}\right)=e\left(h,\tilde{X}\right)e\left(m_{n},\tilde{g}\right)\prod_{i=1}^{n-1}e\left(m_{i},{\tilde{Y}}_{i}\right)$ . If at least one of these equations is not satisfied, the algorithm outputs 0; otherwise, it outputs 1.

### 3.5. Zero-Knowledge Signature of Knowledge

The ZKSoK protocol [22] for an NP-relation R is composed of the following algorithms to ensure knowledge of the language $L_{R}=\left\{y:\exists x,\left(x,y\right)\in R\right\}$ .

$•\;\mathrm{Gen}(1^{\lambda })$ , which returns a public parameter pp when provided a security parameter λ ;

•Sign(m,x,y) , which returns a ZKSoK Π=ZKSoK{x:(x,y)∈R} when given a message *m* and a relation (x,y)∈R ;

•Verify(m,Π,y) , which, when given a message *m*, ZKSoK Π , and statement *y*, returns 1 if Π is valid and 0 otherwise.

## 4. System and Security Model

### 4.1. System Model

**Figure 1:**
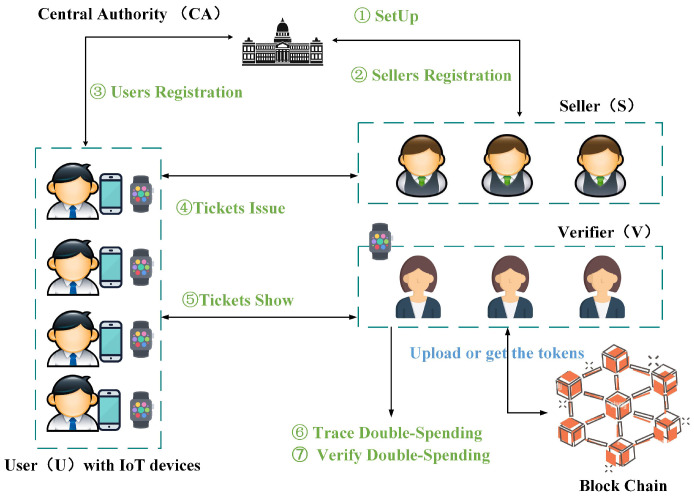
E-ticket system.

Electronic ticketing systems involve five entities, as presented in Figure 1: the Central Authority (CA), Ticket Seller (S), User (U) with IoT device (such as smartphones), Blockchain ( BC ), and Ticket Verifier (V). This section describes each individual function in turn.

• CA bears the responsibility of being the globally trusted entity that establishes the electronic ticketing system (step ➀). Here, the CA creates policy set for purchasing tickets. In addition, the CA must offer registration services to ticket sellers (step ➁) and users (step ➂).

• S is an independent ticket seller who is required to register with CA in order to participate in the ticketing system (step ➁). During the ticket purchasing process, S is responsible for verifying each user’s credentials and issuing tickets based on the ticket purchasing policy set forth by CA (step ➃).

• U has a designated set of attributes stored in their IoT device that must be registered with CA in order to participate in the ticketing system (step ➂). When purchasing a ticket from S, U is only required to disclose a subset of their attributes that satisfy the ticket purchasing policy selected by S (step ➃). Subsequently, U presents the ticket to V in an anonymous manner (step ➄).

• The blockchain BC acts as an immutable database in the electronic ticket system; using the decentralized features of the blockchain, the verifier can upload all valid token information. Any participant can then query the presentation tokens.

• V offers ticket verification services to all users, and has the ability to detect instances of double-spending. Upon receiving verifiable data from users, V verifies the authenticity of their tickets (step ➄) and uploads the token to the blockchain while simultaneously detecting any attempts at double-spending. If a double-spending ticket is identified, V traces the corresponding user’s public key (step ➅) and generates double-spending blaming information.

• Any participant can download the token information from the blockchain and verify the correctness of the double-spending tracing based on the double-spending blaming information generated by V (step ➆).

### 4.2. Formal Definition

The notations used in the system are listed in Table 1, and the algorithms are defined formally below.

$•\;\mathrm{Setup}\left(1^{\lambda }\right)\to \left(msk,pp,P\right)$ : the algorithm is executed by CA, which takes a security parameter $1^{\lambda }$ as input and returns a secret key msk , system parameter pp , and set of ticket purchasing policies P .

•SKeyGen(pp)→(ssk,spk) : the algorithm is executed by S, which takes a system parameter pp as input and returns a private key ssk and corresponding public key spk as output.

•UKeyGen(pp)→(usk,upk) : the algorithm is executed by U, which takes a system parameter pp as input and returns a private key usk and corresponding public key upk as output.

•SReg(S(ssk,spk,pp)↔CA(msk,spk,pp))→ $\left(cred_{s},⊥\right)$ : to obtain a credential for a given public key spk , S and CA engage in an interactive algorithm. S provides (ssk,spk) and pp as inputs, while CA provides msk , spk , and pp as inputs. If the algorithm executes successfully, a credential $cred_{s}$ is issued to S. If the algorithm fails to execute, ⊥ is returned as the output.

$•\;\mathrm{UReg}(U(usk,upk,A,pp)↔\mathrm{CA}(msk,upk,pp))\to (cred_{u},$ ⊥) : U and CA engage in an interactive algorithm to obtain a credential for the attribute set $A={\left\{a_{i}\right\}}_{i=1}^{N}$ . U provides (usk,upk) , A , and pp as inputs, while CA provides msk , upk , and pp as inputs. If the algorithm executes successfully, a credential $cred_{u}$ is issued to U. If the algorithm fails to execute, ⊥ is returned as output.

$•\;\mathrm{Issue}(U\left(usk,cred_{u},D,A,pp\right)↔S(ssk,cred_{s},$ $pp,P))\to \left(\left(tkt,VP_{tkt}\right),b\right)$ : the algorithm for obtaining an anonymous ticket involves an interaction between U and S. To initiate this process, U provides usk , $cred_{u}$ , D , A , and pp as inputs, while S provides ssk , $cred_{s}$ , pp , and P as inputs. Here, we define $D={\left\{a_{i}\right\}}_{i\in D},D\subseteq \left[N\right]$ for convenience. U must then provide proof that a subset D of their attributes A has been certified and that D∈P in accordance with the ticket purchasing policy selected by S. The final output of the algorithm consists of a bit *b* indicating the validity of $cred_{u}$ as well as an anonymous ticket tkt and its associated valid period $VP_{tkt}$ for U.

$•\;\mathrm{Show}(U\left(usk,tkt,VP_{tkt},spk,pp\right)↔V\left(spk,pp\right))$ →((dsid, dstrace),b) : this algorithm relies on the interaction between U and V. U accepts usk , tkt , $VP_{tkt}$ , spk , and pp as inputs, while V only takes spk and pp . When the algorithm is completed, V uploades the token to BC and produces a bit *b* with a value of either 1 or 0 (1 indicates that tkt is valid and that $VP_{tkt}$ falls within the validity period, whereas 0 indicates the opposite) along with a double-spending identity dsid and double-spending trace information dstrace .

$•\;\mathrm{DSTrace}\left(dsid,dstrace,\bar{dsid},\bar{dstrace},spk,pp\right)\to ((dsblame,$ $upk^{\prime }),⊥)$ : V can execute this algorithm under the condition that the double-spending identity of two tickets is identical ( $dsid=\bar{dsid}$ ), which can be correctly verified by traversing the token information in BC . By taking two trace information dstrace and $\bar{dstrace}$ as input, V can deterministically generate two outputs: the unique public key $upk^{\prime }$ of the double-spending user, and the double-spending blaming information ( dsblame if successful or ⊥ if unsuccessful).

$•\;\mathrm{VerifyDS}(dsblame,upk^{\prime })\to b$ : any party can operate this algorithm. With dsblame and $upk^{\prime }$ as inputs, it generates an output of b=1 if dsblame proves that $upk^{\prime }$ has double-spending and b=0 otherwise.

Note that instead of embedding the user ID as an attribute in the user credential, in our e-ticketing system we use each user’s public key as that user’s unique identifier. This is because in our system each user has a unique public key. When the DSTrace algorithm is executed, the user’s ID can always be determined in the real world according to the public key issued by V and the credential information maintained by CA.

**Definition** **1.** *The correctness of the e-ticket system depends on two conditions: (1) the tickets produced by the* *Issue* *algorithm must be verifiable by the* *Show* *algorithm, and (2) the* *DSTrace* *algorithm must be able to track users who attempt double-spending behavior. The formal definition of correctness can be found in Supplementary Material Section S4.*

### 4.3. Security Model

We assume that the central authority in the system, CA, is fully trustworthy. The ticket seller, S, is honest enough to issue tickets to users according to a specific purchase policy but may attempt to obtain users’ undisclosed attributes and real IDs. The user, U, may forge tickets, attempt to spend them twice, or transfer them illegally. The ticket verifier is honest in verifying tickets and detecting users who attempt to spend an electronic ticket more than once, but may attempt to obtain the undisclosed attributes and real IDs of honest users.

The e-ticket system should satisfy the following security requirements: unforgeability of user credentials and tickets, unlinkability of honest users, framing resistance of honest users, and non-transferability of tickets. The security model is defined following the works in [20,23,43,47], and we provide formal definitions of security requirements.

The following global variables and oracles are used in all security definitions.

**Global Variables**.

HU : the set of honest users’ identities; CU : the set of corrupt users identities; ( UPK , USK ): the list of users’ public and secret keys; ( ${\mathrm{CRED}}_{U}$ , ${\mathrm{ATTR}}_{U}$ , ${\mathrm{CID}}_{U}$ ): the list of user credentials, user attributes sets, and user identities; ( TKT , ${\mathrm{VP}}_{tkt}$ , ${\mathrm{TID}}_{U}$ , ${\mathrm{TID}}_{S}$ ): the list of tickets, valid period of tickets, user identities, and seller identities; HS : the set of honest sellers’ identities; CS : the set of corrupt sellers’ identities; ( SPK , SSK ): the list of sellers’ public and secret keys; ( ${\mathrm{CRED}}_{S}$ , ${\mathrm{CID}}_{S}$ ): the list of sellers’ credentials and identities.

**Oracles**.

$•\;O_{HU}\left(i\right)$ : an oracle that can be used to generate keys for an honest user *i*. If i∈HU or i∈CU , then it returns ⊥; otherwise, it creates honest user *i* by running (USK[i],UPK[i]) ←UKeyGen(·) . It adds *i* to HU and returns UPK[i] .

$•\;O_{HS}\left(j\right)$ : an oracle that can be used to generate keys for an honest seller *j*. If j∈HS or j∈CS , then it returns ⊥; otherwise, it creates honest seller *j* by running (SSK[j],SPK[j])←SKeyGen(·) . It adds *j* to HS and returns SPK[j] .

$•\;O_{CU}\left(i,upk\right)$ : an oracle that can (optionally) be used to corrupt an honest user *i* with the public key upk . If i∈CU , then it returns ⊥. If i∈HU , then it removes *i* from HU and adds *i* to CU ; it searches *u* to fulfill the condition ${\mathrm{CID}}_{U}\left[u\right]=i$ and returns ( USK[i] , ${\mathrm{ATTR}}_{U}\left[u\right]$ , ${\mathrm{CRED}}_{U}\left[u\right]$ ). Otherwise, it adds *i* to CU and sets UPK[i]←upk .

$•\;O_{CS}\left(j,spk\right)$ : an oracle that can (optionally) be used to corrupt an honest seller *j* with the public key spk . If j∈CS , then it returns ⊥. If j∈HS , then it removes *j* from HS , adds *j* to CS , and returns SSK[j] . Otherwise, it adds *j* to CS and sets SPK[j]←spk .

$•\;O_{UReg}\left(i,A\right)$ : an oracle that can be used to issue a credential for an honest user *i* with the attribute set A . If i∉HU , it returns ⊥. Otherwise, it issues a credential to *i* by running UReg(U(USK[i],UPK[i],A,pp)↔CA(msk, $\mathrm{UPK}\left[i\right],pp))\to cred_{u}$ and appends $\left(cred_{u},A,i\right)$ to ( ${\mathrm{CRED}}_{U}$ , ${\mathrm{ATTR}}_{U}$ , ${\mathrm{CID}}_{U}$ ).

$•\;O_{SReg}\left(j\right)$ : an oracle that can be used to issue a credential for an honest seller *j*. If j∉HS , it returns ⊥; otherwise, it issues a credential to *j* by running SReg(S(msk,SPK[j], $\mathrm{SPK}\left[j\right],pp)↔\mathrm{CA}\left(\mathrm{SSK}\left[j\right],pp\right))\to cred_{s}$ and appends $(cred_{s},$ j) to ( ${\mathrm{CRED}}_{S}$ , ${\mathrm{CID}}_{S}$ ).

$•\;O_{Iss}\left(i,j,D\right)$ : an oracle that can be used to play an honest seller *j* issuing a ticket to an honest user *i* with the disclosed attributes set $D={\left\{a_{i}\right\}}_{i\in D}$ . If i∉HU or j∉HS , it returns ⊥; otherwise, it searches *u* and *v* to fulfill conditions ${\mathrm{CID}}_{U}\left[u\right]=i$ and ${\mathrm{CID}}_{S}\left[v\right]=j$ , then *j* issues a ticket to *i* by running $\mathrm{Issue}(U\left(\mathrm{USK}\left[i\right],{\mathrm{CRED}}_{U}\left[u\right],D,{\mathrm{ATTR}}_{U}\left[u\right],pp\right)$ ↔S( $\mathrm{SSK}\left[j\right],{\mathrm{CRED}}_{S}\left[v\right],pp,P))\to \left(\left(tkt,VP_{tkt}\right),b\right)$ . If b=0 , it returns ⊥; otherwise, it appends ( tkt , $VP_{tkt}$ , *i*, *j*) to ( TKT , ${\mathrm{VP}}_{tkt}$ , ${\mathrm{TID}}_{U}$ , ${\mathrm{TID}}_{S}$ ).

$•\;O_{{Iss}_{U}}\left(i,j,D\right):$ an oracle that can be used to play a curious seller *j* issuing a ticket to an honest user *i* with the disclosed attributes set D . If i∉HU or j∉CS , it returns ⊥; otherwise, it searches *u* to fulfill the condition ${\mathrm{CID}}_{U}\left[u\right]=i$ and runs $\mathrm{Issue}(U(\mathrm{USK}\left[i\right],{\mathrm{CRED}}_{U}\left[u\right],D,$ ${\mathrm{ATTR}}_{U}\left[u\right],pp)↔A\left(j,\cdot \right))\to \left(\left(tkt,VP_{tkt}\right),b\right)$ , where the seller’s side is executed by the adversary. If b=0 , it returns ⊥; otherwise, it appends ( tkt , $VP_{tkt}$ , *i*, *j*) to ( TKT , ${\mathrm{VP}}_{tkt}$ , ${\mathrm{TID}}_{U}$ , ${\mathrm{TID}}_{S}$ ).

$•\;O_{{Iss}_{S}}\left(j,i,D\right)$ : an oracle that can be used to play an honest seller *j* issuing a ticket to a malicious user *i* with the disclosed attributes set D . If i∉CU or j∉HS , it returns ⊥; otherwise, it searches *v* to fulfill the condition ${\mathrm{CID}}_{S}\left[v\right]=j$ and runs $\mathrm{Issue}\left(A\left(i,\cdot \right)↔S\left(\mathrm{SSK}\left[j\right],{\mathrm{CRED}}_{S}\left[v\right],pp,P\right)\right)\to \left(\left(tkt,VP_{tkt}\right),b\right)$ , where the user’s side is executed by the adversary. If b=0 , it returns ⊥; otherwise, it appends ( tkt , $VP_{tkt}$ , *i*, *j*) to ( TKT , ${\mathrm{VP}}_{tkt}$ , ${\mathrm{TID}}_{U}$ , ${\mathrm{TID}}_{S}$ ).

$•\;O_{Shw}\left(i,j\right)$ : an oracle that can be used to play a malicious verifier verifying a ticket for an honest seller user *i*. If i∉HU , it returns ⊥; otherwise, it searches *u* to fulfill conditions ${\mathrm{TID}}_{U}\left[u\right]=i$ and ${\mathrm{TID}}_{S}\left[u\right]=j$ and runs Show(U(USK[i], $\mathrm{TKT}\left[u\right],{\mathrm{VP}}_{tkt}\left[u\right],pp)↔A\left(\mathrm{SPK}\left[j\right],\cdot \right))\to \left(\left(dsid,dstrace\right),b\right)$ , where the verifier’s side is executed by the adversary. If b=0 , it returns ⊥; otherwise, it returns (dsid,dstrace) .

We define the security model of the PriTKT system as follows.

**Unforgeability**. Unforgeability can protect honest sellers and verifiers from malicious users. It guarantees that users cannot forge credentials in the Issue algorithm or tickets in the Show algorithm. An adversary can interact with the CA and honest seller oracles as a corrupted user to model this property. The adversary wins when they can forge a credential or a ticket of either an honest or an unregistered user in the Issue or Show algorithm. Unforgeability is defined by dividing it into credential unforgeability and ticket unforgeability.

**Definition** **2.** *Experiment $Exp^{uf_{cred}}$ in Pse. 1 defines the unforgeability of credentials. The users’ credentials are considered unforgeable if any PPT adversary A can access the oracle $O=\{O_{HU}\left(i\right),O_{CU}\left(i\right),O_{HS}\left(j\right),O_{UReg}\left(i,A\right),$ $O_{SReg}\left(j\right),O_{Iss}\left(i,j,D\right),$ $O_{{Iss}_{S}}\left(j,i,D\right),O_{Shw}\left(i,j\right)\}$ . A negligible function ϵ(λ) exists such that*

$$\begin{array}{c} Adv^{uf_{cred}}=\left|\mathrm{Pr}\left[Exp^{uf_{cred}}\left(A,\lambda \right)\right]=1\right|⩽\epsilon \left(\lambda \right). \end{array}$$

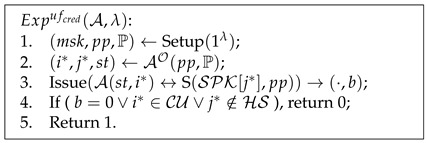

  Pseudocode 1. Unforgeability of Credentials

**Definition** **3.** *The experiment $Exp^{uf_{tkt}}$ in Pse. 2 defines the unforgeability of tickets. In order for users’ tickets to be considered unforgeable, it must be the case that any PPT adversary, denoted as A , who has the oracle O that contains $O_{HU}\left(i\right)$ , $O_{CU}\left(i\right)$ , $O_{HS}\left(j\right)$ , $O_{UReg}\left(i,A\right)$ , $O_{SReg}\left(j\right)$ , $O_{Iss}\left(i,j,D\right)$ , $O_{{Iss}_{S}}\left(j,i,D\right)$ , and $O_{Shw}\left(i,j\right)$ , will have a negligible function ϵ(λ) such that*

$$\begin{array}{c} Adv^{uf_{tkt}}=\left|\mathrm{Pr}\left[Exp^{uf_{tkt}}\left(A,\lambda \right)\right]=1\right|⩽\epsilon \left(\lambda \right). \end{array}$$

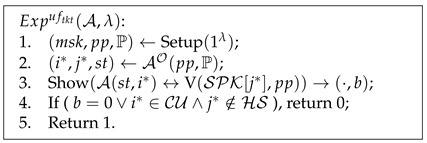

  Pseudocode 2. Unforgeability of Tickets

**Unlinkability**. Unlinkability is necessary to safeguard honest users from inquisitive sellers and verifiers. Its primary purpose is to prevent an adversary from controlling a corrupt seller (who also verifies) from associating a specific credential with a particular user in the Issue algorithm or from tying a specific ticket to a particular user in the Show algorithm. To formally define this property, we allow the adversary to interact with the CA and honest user oracles playing the part of the corrupted seller. In the subsequent challenge phase, the adversary can invoke extra interactions and attempt to determine which user they are interacting with. If they correctly guess the user, the adversary wins. To define this property, we divide unlinkability into unlinkability of credentials and unlinkability of tickets.

**Definition** **4.** *The experiment $Exp^{ano_{cred}-b}$ depicted in Pse. 3 defines the unlinkability of credentials. We consider users’ credentials to be unlinkable if, for any PPT adversary, A with access to the oracle $O=\{O_{HU}\left(i\right),O_{HS}\left(j\right),O_{CS}\left(j\right),O_{UReg}\left(i,A\right),$ $O_{SReg}\left(j\right),O_{Iss}\left(i,j,D\right),$ $O_{{Iss}_{U}}\left(i,j,D\right)$ , $O_{Shw}\left(i,j\right)\}$ there exists a negligible function ϵ(λ) such that*

$$\begin{array}{c} Adv^{ano_{cred}}=|\mathrm{Pr}\left[Exp^{ano_{cred}-1}\left(A,\lambda \right)=1\right]-\\ \mathrm{Pr}\left[Exp^{ano_{cred}-0}\left(A,\lambda \right)=1\right]|⩽\epsilon \left(\lambda \right). \end{array}$$

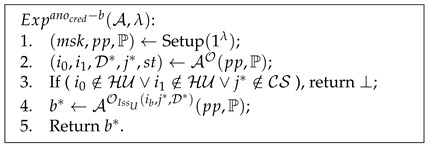

  Pseudocode 3. Anonymity of Credentials

**Definition** **5.** *Experiment $Exp^{ano_{tkt}-b}$ in Pse. 4 defines the unlinkability of tickets. Users’ tickets are unlinkable if any PPT adversary A with the oracle $O=\{O_{HU}\left(i\right),O_{HS}\left(j\right),O_{CS}\left(j\right),$ $O_{UReg}\left(i,A\right),O_{SReg}\left(j\right),$ $O_{Iss}\left(i,j,D\right),O_{{Iss}_{U}}\left(i,j,D\right),O_{Shw}\left(i,j\right)\}$ cannot associate a specific ticket with a particular user. There exists a negligible function ϵ(λ) such that*

$$\begin{array}{c} Adv^{ano_{tkt}}=|\mathrm{Pr}\left[Exp^{ano_{tkt}-1}\left(A,\lambda \right)=1\right]-\\ \mathrm{Pr}\left[Exp^{ano_{tkt}-0}\left(A,\lambda \right)=1\right]|⩽\epsilon \left(\lambda \right). \end{array}$$

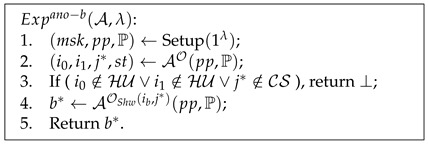

  Pseudocode 4. Anonymity of Tickets

**Framing Resistance**. In defining framing resistance, we follow the idea of Bobolz et al. [23]. Framing resistance ensures that corrupt sellers cannot falsely accuse honest users of double spending. To model this property, we allow the adversary to interact with the CA and the oracles of the honest users in the role of a corrupt seller. Honest users will not engage in double spending. The adversary outputs a double-spending blame information dsblame . If there is an honest user’s public key $upk^{\prime }$ that verifies dablame and $upk^{\prime }$ with VerifyDS, then the adversary wins.

**Definition** **6.** *The framing resistance property is defined by the experiment $Exp^{fr}$ in Pse. 5. The e-ticket system is framing-resistant if, for any PPT adversary A having access to the oracle $O=\{O_{HU}\left(i\right),O_{HS}\left(j\right),O_{CS}\left(j\right),O_{UReg}\left(i,A\right),$ $O_{SReg}\left(j\right),$ $O_{Iss}\left(i,j,D\right),O_{{Iss}_{U}}\left(i,j,D\right),$ $O_{Shw}\left(i,j\right)\}$ , there is a negligible function ϵ(λ) such that*

$$\begin{array}{c} Adv^{fr}=\left|\mathrm{Pr}\left[Exp^{fr}\left(A,\lambda \right)=1\right]\right|⩽\epsilon \left(\lambda \right). \end{array}$$

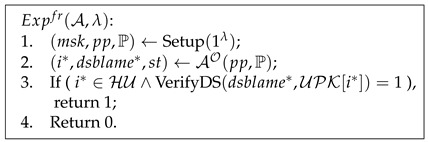

  Pseudocode 5. Framing Resistance

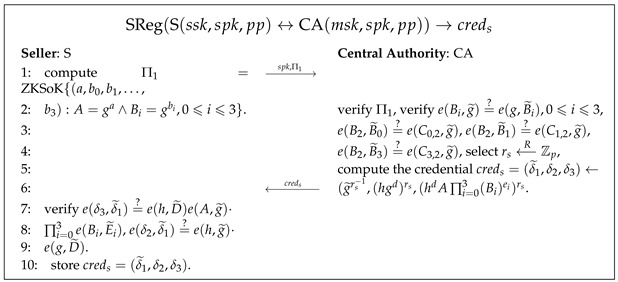

  Pseudocode 6. Seller Registration Algorithm

## 5. Our Construction

### 5.1. Overflow of PriTKT

PriTKT’s workflow, illustrated in Figure 1, operates in the following manner. The CA initializes the system by issuing a system parameter pp and a set of ticket purchase policies P (Setup, step ➀). Upon joining the system, the Seller (S) creates a private–public key pair ( ssk,spk ) by running SKeyGen, authenticates to the CA, and obtains their public key credentials $cred_{s}$ (SReg, step ➁). The User (U) generates the private–public key pair ( usk,upk ) using UKeyGen, authenticates to the CA, and obtains their attribute-based credentials $cred_{u}$ (UReg, step ➂). To obtain a ticket, U verifies their identity to S by providing a disclosure proof for selective attributes (including attributes D⊂A ) and S generates a ticket tkt for U (Issue, step ➃). Upon presenting the ticket, U anonymously proves its validity to Verifier (V) and discloses its validity period $VP_{tkt}$ and double-spending identity dsid (Show, step ➄). V can traverse the token information in the blockchain and verify whether a double-spending event has occurred. In case of attempted double-spending, V can trace U’s public key $upk^{\prime }$ using a ticket tkt and generate blame information (DSTrace, step ➅). Using this blame information, any entity involved can verify the accuracy of the double-spending trace (VerifyDS, step ➆).

### 5.2. High-Level Overview

In this section, we present a specific implementation of the e-ticket system defined in Section 4. The main challenge in developing the e-ticket system is creating efficient and unlinkable ticket issuance and show algorithms. The classical solution used in this regard consists of zero-knowledge proofs that prove the knowledge of hidden attributes, where those attributes are signed by a certification authority or a signer. Han et al. [18] have proposed an attribute-based e-ticketing system using this approach, and to date this remains the only such system. Han et al.’s system includes a privacy-preserving e-ticketing system with attribute-based credentials, BBS signatures, and NIZK. While this solution fulfills privacy requirements, the system is very costly, with the Issue algorithm’s computational complexity being O(N) , where *N* is the number of user attributes. This significantly limits its practical use. Another strategy is to use specific signatures to prove knowledge of a subset of user attributes. The URS signature [20,44] is a constant-size signature that proves *K* out of *N* attributes, and its computational complexity is O(N−K) for the prover and O(K) for the verifier. Sanders [20] has proposed an extremely efficient URS scheme that can easily unlink without cost. Therefore, we have chosen to use Sanders’ URS scheme to create the attribute credentials $cred_{u}$ and tickets tkt . URS ensures that the displayed credentials cannot be linked and supports the proof of attribute disclosure. The most important advantage of URS is that it uses randomization proof technology, avoiding the need for complex zero-knowledge proofs.

Because the private key of S is used to issue tickets to U, S generates the public–private key pair of URS, and the CA must issue the credential for the public key of S. SPS [21] allows us to effectively implement credential issuance for URS public keys.

The tickets are designed as single-use tickets, with a need to prevent double-spending by users. Moreover, in the case of double-spending, a user’s public key needs to quickly be traced. To meet these requirements, we use the “Schnorr trick” [23]. When presenting tickets, a user discloses their double-spending identity dsid to the verifier; the verifier can instantly detect double-spending if a similar identity was used earlier. Specifically, every time a user shows a ticket, they create a challenge value $c^{\prime }$ and calculate $s^{\prime }=dsrnd+usk\cdot c^{\prime }$ , employing the “Schnorr trick”. When double-spending occurs, the user is required to show $s^{\prime }=dsrnd+usk\cdot c^{\prime }$ in the first show and ${\bar{s}}^{\prime }=dsrnd+usk\cdot {\bar{c}}^{\prime }$ in the second show. The zero-knowledge proof of knowledge ensures that usk and dsrnd are the same for both shows, while $\left(s^{\prime },{\bar{s}}^{\prime },c^{\prime },{\bar{c}}^{\prime }\right)$ enables the verifier to calculate usk . If a user does not double-spend, usk remains perfectly hidden in $s^{\prime }$ (because dsrnd is only used once) and each displayed dsid is simply a random identity.

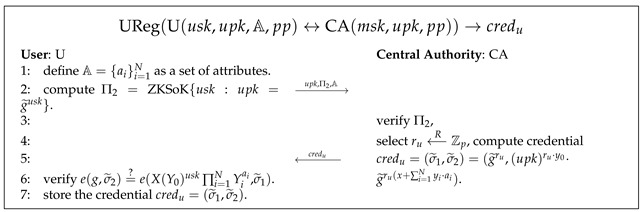

  Pseudocode 7. User Registration Algorithm

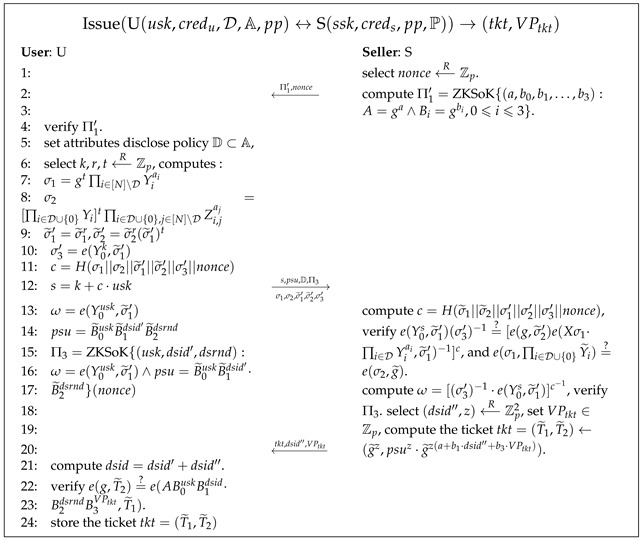

  Pseudocode 8. Issue Tickets Algorithm

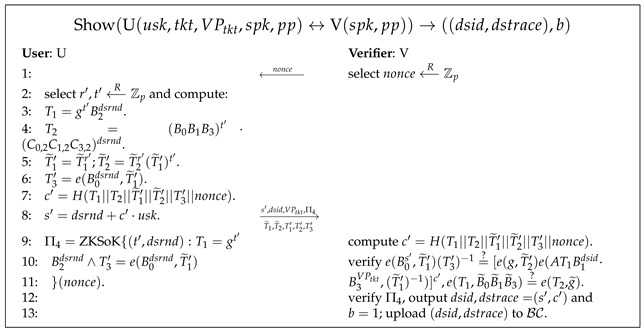

  Pseudocode 9. Show Tickets Algorithm

### 5.3. Concrete Construction

$\mathrm{Setup}\left(1^{\lambda }\right)\to \left(msk,pp,P\right)$ : CA takes the security parameter $1^{\lambda }$ as input to create the system master private key msk , public parameters pp , and a ticket purchasing policy set P . CA defines a ticket purchasing policy set P and generates Type-III bilinear pair parameters $\left(G_{1},G_{2},G_{T},g,\tilde{g},p,e\right)$ . CA selects $\left(x,y_{0},y_{1},\ldots ,y_{N}\right)\overset{\;R\;}{\leftarrow }Z_{p}^{N+2}$ , $\left(d,e_{0},e_{1},\ldots ,e_{3}\right)$ $\overset{\;R\;}{\leftarrow }Z_{p}^{5}$ , $h\overset{\;R\;}{\leftarrow }G_{1}$ , and computes $X=g^{x},Y_{i}=g^{y_{i}},{\tilde{Y}}_{i}={\tilde{g}}^{y_{i}},Z_{i,j}=g^{y_{i}\cdot y_{j}}$ for 0⩽i≠j⩽N . In addition, CA computes $\tilde{D}={\tilde{g}}^{d}$ and ${\tilde{E}}_{i}={\tilde{g}}^{e_{i}},0⩽i⩽3$ . CA then outputs $msk=\{x,y_{0},y_{1},\ldots ,y_{N},d,e_{0},e_{1},\ldots ,e_{3}\}$ and $pp=\{g,\tilde{g},h,X,Y_{0},\ldots ,Y_{N},{\tilde{Y}}_{0},\ldots ,{\tilde{Y}}_{N},$ ${\left\{Z_{i,j}\right\}}_{0⩽i\neq j⩽N},$ $\tilde{D},{\tilde{E}}_{0},\ldots ,{\tilde{E}}_{3}\}$ .

SKeyGen(pp)→(ssk,spk) : S takes public parameters pp as input to generate its secret key ssk and public key spk . S selects $\left(a,b_{0},b_{1},\ldots ,b_{3}\right)$ $\overset{\;R\;}{\leftarrow }Z_{p}^{5}$ and computes $A=g^{a}$ , $B_{i}=g^{b_{i}}$ , ${\tilde{B}}_{i}={\tilde{g}}^{b_{i}}$ , $C_{0,2}=g^{b_{0}\cdot b_{2}}$ , $C_{1,2}=g^{b_{1}\cdot b_{2}}$ , and $C_{3,2}=g^{b_{3}\cdot b_{2}}$ , where 0⩽i⩽3 . S outputs $ssk=\{a,b_{0},b_{1},\ldots ,b_{3}\}$ and $spk=\{A,B_{0},\ldots ,B_{3},{\tilde{B}}_{0},\ldots ,{\tilde{B}}_{3},C_{0,2},C_{1,2},C_{3,2}\}$ .

UKeyGen(pp)→(usk,upk) : U takes public parameters pp as input to generate the secret key usk and public key upk . U selects $usk\overset{\;R\;}{\leftarrow }Z_{p}$ and computes $upk={\tilde{g}}^{usk}$ .

$\mathrm{SReg}\left(S\left(ssk,spk,pp\right)↔\mathrm{CA}\left(msk,spk,pp\right)\right)\to cred_{s}$ : As shown in Pse. 6, S interacts with CA to generate the seller’s public key credential $cred_{s}$ . S sends CA the public key spk along with a ZKSoK of ssk ( $\Pi_{1}$ ) to prove that S knows the secret key ssk . S should authenticate to CA and provide (online or offline) evidence to demonstrate that it can operate as a seller. If $\Pi_{1}$ is verified as valid and the authentication is accepted, CA computes an SPS signature $cred_{s}$ as the credential of the public key spk . $cred_{s}$ is then sent back to S, who uses its private and public keys and their associated credential to verify that CA has authorized it as a seller.

$\mathrm{UReg}(U(usk,upk,A,pp)↔\mathrm{CA}(msk,upk,pp))\to cred_{u}$ : as shown in Pse. 7, U interacts with CA to generate the user’s attribute credential $cred_{u}$ . U sends CA the public key upk along with a ZKSoK of usk ( $\Pi_{2}$ ) and a set of attributes $A={\left\{a_{i}\right\}}_{i=1}^{N}$ which allow U to purchase tickets. If $\Pi_{2}$ is verified as valid and the attributes are authentic, CA computes a URS signature $cred_{u}$ as the credential of the public key upk and user attributes A . $cred_{u}$ is sent back to U, who uses it to verify that they are now a legitimate user and that their attributes have been signed by CA.

$\mathrm{Issue}(U\left(usk,cred_{u},D,A,pp\right)↔S\left(ssk,cred_{s},pp,P\right))$ $\to (tkt,VP_{tkt})$ : as shown in Pse. 8, U interacts with S to obtain the ticket tkt . To prevent S from collecting user information maliciously, S computes the signature of knowledge $\Pi_{1}^{\prime }$ to prove to the user that it has been authenticated by CA. To prevent replay attacks, S chooses a random nonce to send to U. U anonymously proves to S that they have been certified by CA as a legitimate user and selects an attributes set D to disclose in order to satisfy a ticket purchasing policy selected by S from the policy set defined by CA. U then generates a new Pedersen commitment psu which commits a private key usk , double-spending identity $dsid^{\prime }$ , and double-spending random dsrnd . U constructs a ZKSoK ( $\Pi_{3}$ ) to prove that U knows the knowledge of $\left(usk,dsid^{\prime },dsrnd\right)$ and that psu and $cred_{u}$ have the same usk . If $\Pi_{3}$ is verified as valid, S can update the Pedersen commitment psu homomorphically without knowing the opening, then produces the ticket tkt , contributes to double-spending identity $dsid^{\prime \prime }$ of S, and clarifies the ticket’s valid period $VP_{tkt}$ . S then sends tkt , $dsid^{\prime \prime }$ , and $VP_{tkt}$ to U, who uses them along with U’s private key to verify the validity of the ticket.

$\mathrm{Show}(U\left(usk,tkt,VP_{tkt},spk,pp\right)↔V\left(spk,pp\right))$ →((dsid, dstrace),b) : as shown in Pse. 9, U interacts with V to show the ticket tkt . To prevent replay attacks, V first chooses a random nonce to send to U. U anonymously proves the legitimacy of tkt to V and discloses dsid and $VP_{tkt}$ . Then, U computes the challenge $c^{\prime }$ and $s^{\prime }=dsrnd+usk\cdot c^{\prime }$ to enable V to reveal the user’s public key in case of double-spending. Finally, U needs to compute $\Pi_{4}$ to ensure that the dsrnd in the tkt is the same as in $s^{\prime }$ . If $\Pi_{4}$ is verified, V checks all tickets in the history with the same dsid to detect whether the ticket has been double-spent. If not, V uploads (dsid,dstrace) to BC and outputs b=1 ; otherwise, b=0 . Then, V traverses the token information in the blockchain and verifies whether a double-spending event has occurred. In case of double-spending being detected, V outputs dsid to link double-spending tickets and uses dstrace to trace the double-spending user’s public key.

$\mathrm{DSTrace}\left(dsid,dstrace,\bar{dsid},\bar{dstrace},spk,pp\right)\to (dsblame,$ $upk^{\prime })$ : if U spends the same ticket a second time, this algorithm can be operated by V. If U only spends their ticket once, then usk is perfectly hidden. However, in the case of double-spending V can detect whether the dsid of the current ticket is the same as the $\bar{dsid}$ of a ticket that was spent before. This allows V to compute the private key usk of a double-spending user based on the fact that double-spending of the same ticket involves the same dsid and two different challenges $\left(c^{\prime },{\bar{c}}^{\prime }\right)$ with $s^{\prime }=dsrnd+usk\cdot c^{\prime }$ , ${\bar{s}}^{\prime }=dsrnd+usk\cdot {\bar{c}}^{\prime }$ , allowing V to extract usk with overwhelming probability by parsing $\left\{dsid,dstrace=\left(s^{\prime },c^{\prime }\right)\right\}$ and $\left\{\bar{dsid},\bar{dstrace}=\left({\bar{s}}^{\prime },{\bar{c}}^{\prime }\right)\right\}$ . If $dsid=\bar{dsid}$ , $dsblame=\frac{s^{\prime }-{\bar{s}}^{\prime }}{{\bar{c}}^{\prime }-c^{\prime }}$ is output and $upk^{\prime }={\tilde{g}}^{dsblame}$ ; otherwise, ⊥ is output. It should be noted that because the user generates $c^{\prime }$ by using a random nonce selected by V every time a ticket is shown, there is an overwhelming probability that generation of two different $c^{\prime }$ will be forced when a ticket is double-spent.

$\mathrm{VerifyDS}(dsblame,upk^{\prime })\to b$ ; given double-spending blaming information dsblame and a public key upk , this algorithm outputs b=1 if $upk^{\prime }={\tilde{g}}^{dsblame}$ and b=0 otherwise.

The details of the zero-knowledge signature of knowledge of the proposed system are shown in Supplemental Material Section S3.

## 6. Security Analysis

In Supplementary Material Section S1, we analyze the correctness of the proposed system. To formalize that our construction from Section 4.2 satisfies all the desired security guarantees defined in Section 3.2, we define the following theorems. Let $\Pi_{1},\Pi_{2},\Pi_{3},\Pi_{4}$ be ZKSoKs. See Supplementary Material Section S2 for the formal proofs.

**Theorem** **1.** *In the PriTKT system, the user’s credential is unforgeable if the DL assumption holds in $G_{2}$ and if the URS is unforgeable.*

**Theorem** **2.** *In the PriTKT system, the user’s tickets are unforgeable if the DL assumption holds in $G_{2}$ and if the URS is unforgeable.*

**Theorem** **3.** *In the PriTKT system, the user’s credential is unlinkable if the DDH assumption holds in $G_{2}$ .*

**Theorem** **4.** *In the PriTKT system, the user’s tickets are unlinkable if the DDH assumption holds in $G_{2}$ .*

**Theorem** **5.** *The PriTKT system is framing-resistant if the DL assumption holds in $G_{2}$ .*

## 7. Performance Analysis

### 7.1. Theoretical Analysis and Comparison

Table 2 presents a detailed comparison of PriTKT and related works, including four e-ticket systems [12,13,14,16], one attribute-based issuance e-ticket system [18], and five attribute-based credential schemes [35,36,39,40,43]. The comparison evaluates each system in terms of formal security proof, double-spending detection, double-spending trace, attribute-based issuance, and attribute disclosure. Formal proof refers to an e-ticketing system’s security verification through formal methods. Double-spending detection ensures that an e-ticket cannot be reused after it has been spent, while double-spending trace allows for the identification of the responsible user when a ticket is spent twice. Attribute-based issuance allows for the issuance of tickets or credentials based on user attributes. Finally, attribute disclosure refers to the method used by users to reveal a subset of their attributes in order to purchase or display tickets. Of the e-ticketing systems included in the study, all those listed in [12,13,14,16,18] feature double-spending detection, while the systems in [14,18] offer double-spending trace and formal security proof. Furthermore, Ref. [18] employs complex zero-knowledge proofs (ZKP) to issue tickets based on attributes, while the attribute-based credential schemes discussed in [35,36,39,40] use ZKP to issue credentials. Lastly, the attribute-based credential schemes in [43] offer formal proof and attribute-based issuance, with the efficiency of attribute disclosure improved by replacing ZKP with SPS-EQ. However, this scheme fails to prove that the user’s hidden attributes satisfy certain requirements. In comparison, PriTKT boasts formal proof, double-spending detection, double-spending trace, attribute-based issuance, and attribute disclosure. The URS signature used in PriTKT avoids the complexity associated with the use of ZKP in [18].

Table 3, Table 4 and Table 5 feature a comparison of the PriTKT system and the only existing e-ticketing system [18] in terms of computation, storage, and communication overhead. The sizes of the elements in the groups $G_{1}$ , $G_{2}$ , $G_{T}$ , and $Z_{p}$ are represented by $|G_{1}|$ , $|G_{2}|$ , $|G_{T}|$ , and $|Z_{p}|$ , respectively. The time costs for exponentiation in groups $G_{1}$ , $G_{2}$ , and $G_{T}$ , and the bilinear pairing maps are denoted by $t_{e_{1}}$ , $t_{e_{2}}$ , $t_{e_{T}}$ , and $t_{p}$ , respectively. The only difference in implementation between these two systems is that PriTKT uses high-efficiency Type-III pairing, where $G_{1}\neq G_{2}$ , while the system presented in [18] uses Type-I pairing with $G_{1}=G_{2}$ .

Table 3, Table 4 and Table 5 show that the system in [18] is inefficient; a ticket issued on an attribute-based credential (Issue*U*) requires at least O(N) operations in order for a user to disclose *K* attributes out of a total of *N* attributes. In addition, it is necessary to prove knowledge of all *N* attributes, which implies that at least O(N) elements must be sent in communications during the execution of the Issue algorithm. Our algorithm avoids this problem, significantly reducing the computational cost to O(N−K) operations and the communication cost to O(K) elements.

In PriTKT, the computational overhead of Setup and the storage overhead of pp are both $O(N^{2})$ . Fortunately, system initialization only needs to be performed once, and the system parameters pp are stored on users’ IoT devices (such as smartphones), which have more than enough storage space. In PriTKT, the user credential $cred_{u}$ , seller credential $cred_{s}$ , and ticket tkt all have constant sizes. While the operations on Issue*U* are related to the number of user attributes, they decrease with the number of disclosed attributes. The operations for Issue*S* depend only on the number of disclosed attributes. The computation and communication overhead of Show and DSTrace are constant in PriTKT.

### 7.2. Experimental Analysis of PriTKT

We further evaluated the performance of the PriTKT system through objective tests. We implemented the system and measured its performance on an Android 9.0 operating system running on a HUAWEI Honor 9i smartphone, which had a Hisilicon Kirin 659 (ARMv8-A) CPU with a clock speed of 2.36 GHz and 1.7 GHz and with 4 GB of RAM.

We performed experiments utilizing MIRACL [48] and Type-III pairing. We used SHA256 to implement the $H:{\left\{0,1\right\}}^{*}\to Z_{p}$ hash functions required by PriTKT (see Pseudocodes 8 and 9). To accurately evaluate the computational and storage/communication overhead of each of PriTKT’s algorithms, we used the Barreto–Naehrig curve (BN-256) [49]. BN-256 was used to test the system’s performance at the AES 100-bit security level, and we compared it to the performance of a scheme [18] using the Security Supersingular Elliptic Curve (SSP-1536) [50] at the same security level.

In practice, the total number of user attributes *N* can reach hundreds, and is usually much larger than the number of a user’s disclosed attributes *K*. For example, a user may have name , id , student , phone , number , occupation , home , address , and many other attributes. If the user buys a student discount ticket, they only need to provide their student attribute. Therefore, we compared the computation and storage/communication costs with a larger number of *N* and a constant number of K=5 .

Table 6, Table 7 and Table 8 compare the computational and storage/communication overheads of all algorithms between PriTKT and [18] at N=50 , where each time result is averaged over 50 iterations.

As shown in Table 6, in PriTKT the Setup algorithm takes 44.2 s. For key generation, UKeyGen and SKeyGen cost 18.1 ms and 143.6 ms, respectively. For user registration, UReg*U* and UReg*CA* cost 642.8 ms and 95.5 ms, respectively. For seller registration, SReg*S* and SReg*CA* cost 1024.3 ms and 1543.2 ms, respectively. The DSTrace algorithm takes 18.5 ms. Most frequently used of e-tickets rely on two algorithms, namely, Issue and Show. When issuing a ticket, the computation overheads of the Issue*U* algorithm are 1971.4 ms for PriTKT and 561,049 ms for the scheme in [18], while the computation overheads of the Issue*S* algorithm are 908.2 ms for PriTKT and 201,506 ms for the scheme in [18]. When showing a ticket, the computation overheads of the Show*U* algorithm are 320.4 ms for PriTKT and 79,544 ms for the scheme in [18], while the computation overheads of the Show*V* algorithm are 804.3 ms for PriTKT and 69,924 ms for the scheme in [18].

As shown in Table 7, the storage of public parameters pp in PriTKT is 348,816 bytes, which is significantly larger than the 2584 bytes required by the scheme in [18]; however, we note that public parameters can be stored in smartphones with sufficient storage space. The other size overheads are similar for both PriTKT and the scheme in [18].

As shown in Table 8, the communication overheads of the Issue*U* algorithm are 1856 bytes for PriTKT and 6640 bytes for the scheme in [18], while the communication overheads of the Show*U* algorithm are 1568 bytes for PriTKT and 1824 bytes for the scheme in [18]. For both the Issue*S* and Show*V* algorithms, the communication overheads of both PriTKT and the scheme in [18] are less than 1000 bytes.

Table 9 and Table 10 compare the computational and communication overheads of the Issue and Show algorithms between PriTKT and the scheme in [18]; the parameters were set to K=5 , while *N* varied from 20 to 100.

Table 9 shows that the computational overhead of the Issue*U* algorithm in both PriTKT and the scheme of Han et al. [18] increases linearly with the number of user attributes. However, PriTKT performs over 250 times more efficiently than the scheme of Han et al. For the Issue*S* algorithm, the computational overhead of both schemes are independent of the number of user attributes. Nonetheless, PriTKT is around 200 times more efficient than the scheme of Han et al. In the case of the Show*U* and Show*V* algorithms, the computational overhead of both schemes are independent of the number of user attributes. However, PriTKT executes around 240 times and 80 times more efficiently in the Show*U* and Show*V* algorithms, respectively, compared to the scheme of Han et al.

As shown in Table 10, the communication overheads of PriTKT are constant for both the Issue and Show algorithms. The communication overheads of the Issue*U*, Issue*S*, Show*U*, and Show*V* algorithms in PriTKT are as low as 1856 bytes, 904 bytes, 1586 bytes and 120 bytes, respectively. In comparison, while the scheme in [18] achieves a constant communication overhead for the Show algorithm, for the Issue*U* algorithm its output size increases linearly with the number of user attributes.

Compared to the state-of-the-art described in Han et al. [18], the above analysis and comparison reveal that PriTKT incurs significantly less computational and communication overhead.

## 8. Conclusions

This paper presents a blockchain-enhanced privacy-preserving e-ticketing system for IoT devices. The proposed system permits users to purchase tickets anonymously by revealing some of their attributes while concealing others. The proposed system presents significantly reduced computational cost and communication overhead compared to state-of-the-art e-ticketing systems, making it suitable for use in IoT devices. Moreover, it utilizes blockchain technology to achieve effective malicious user tracking. The system possesses robust security properties such as unlinkability, unforgeability, non-double spending, non-transferability, and framing resistance. The security properties are formally defined, and we have reduced them to well-known complexity assumptions or the security of proven cryptography primitives. We implemented the algorithms of the e-ticketing system on a smartphone, demonstrating that the system generates significantly lower computational and communication overhead compared to the state-of-the-art.

## Figures and Tables

**Table 1:** Summary of notation.

Notation	Description
$1^{\lambda }/\epsilon \left(\lambda \right)$	security number/negligible function
[N]	set {1,2,…,N}
D	subset of [N]
$x\overset{R}{\leftarrow }S$	*x* is randomly selected from the set S
CA	central authority
S/U/V	ticket seller/user/verifier
BC	the blockchain
P	ticket purchasing policy set
A/D	attributes/disclosed attributes set
N/K	number of user attributes/disclosed attributes
pp/msk	public parameters/master key of the system
usk/upk	secret/public key of U
ssk/spk	secret/public key of S
$cred_{u}/cred_{s}$	credential of U/S
tkt	ticket of U
$VP_{tkt}$	valid period for the ticket of U
dsid/dstrace	double-spending identity/trace infomation
dsblame	double-spending blaming information
*H*	collision resistant hash function
⊥	failed identifier

**Table 2:** Functional comparison with related works.

Scheme	Formal Proof	Double-Spending Detection	Double-Spending Trace	Attribute-Based Issuance	Disclosing Attributes
[12]	×	√	×	×	−
[16]	×	√	×	×	−
[13]	×	√	×	×	−
[14]	√	√	√	×	−
[18]	√	√	√	√	ZKP
[35]	√	−	−	√	ZKP
[36]	√	−	−	√	ZKP
[39]	√	−	−	√	ZKP
[40]	√	−	−	√	ZKP
[43]	√	−	−	√	SPS-EQ
PriTKT	√	√	√	√	URS

√: supported feature; ×: unsupported feature; −: not applicable.

**Table 3:** Computation overhead comparison.

Algorithms	Entity	PriTKT	[18]
Setup	CA	$\left(N^{2}+1\right)t_{e_{1}}+\left(N+5\right)t_{e_{2}}$	$Nt_{e_{1}}$
UKeyGen	User	$t_{e_{2}}$	$t_{e_{1}}$
SKeyGen	Seller	$8t_{e_{1}}+4t_{e_{2}}$	$t_{e_{1}}$
UReg*U*	User	$\left(N+1\right)t_{e_{1}}+1t_{e_{2}}+2t_{p}$	$\left(N+5\right)t_{e_{1}}+\left(N+4\right)t_{p}$
UReg*CA*	CA	$5t_{e_{2}}$	$\left(N+2\right)t_{e_{1}}$
SReg*S*	Seller	$5t_{e_{1}}+10t_{p}$	$4t_{e_{1}}+5t_{p}$
SReg*CA*	CA	$8t_{e_{1}}+3t_{e_{2}}+14t_{p}$	$3t_{e_{1}}$
Issue*U*	User	$\left(2\left(N-K\right)+20\right)t_{e_{1}}+9t_{e_{2}}+5t_{p}$	$\left(N+3K+37\right)t_{e_{1}}+\left(N+3K+21\right)t_{p}$
Issue*S*	Seller	$\left(K+3\right)t_{e_{1}}+7t_{e_{2}}+4t_{e_{T}}+6t_{p}$	$\left(3K+31\right)t_{e_{1}}+Kt_{e_{T}}+\left(2K+17\right)t_{p}$
Show*U*	User	$8t_{e_{1}}+3t_{e_{2}}+2t_{p}$	$24t_{e_{1}}+8t_{p}$
Show*V*	Verifier	$7t_{e_{1}}+2t_{T}+6t_{p}$	$19t_{e_{1}}+8t_{p}$
DSTrace	Verifier	$t_{e_{2}}$	$3t_{e_{1}}$

**Table 4:** Storage complexity comparison.

Variates	Entity	PriTKT	[18]
pp	CA	$\left(N^{2}+3\right)|G_{1}|+\left(N+6\right)\left|G_{2}\right|$	$\left(N+13\right)|G_{1}|$
upk	User	$1|G_{2}|$	$1|G_{1}|$
spk	Seller	$8|G_{1}\left|+4\right|G_{2}|$	$1|G_{1}|$
$cred_{u}$	User	$2|G_{1}|$	$2|Z_{p}\left|+1\right|G_{1}|$
$cred_{s}$	Seller	$2|G_{1}\left|+1\right|G_{2}|$	$2|Z_{p}\left|+1\right|G_{1}|$
tkt	User	$4|Z_{p}\left|+2\right|G_{2}|$	$5|Z_{p}\left|+2\right|G_{1}|$
tok	User	$5|Z_{p}\left|+2\right|G_{1}\left|+2\right|G_{2}\left|+1\right|G_{T}|$	$10|Z_{p}\left|+8\right|G_{1}|$

**Table 5:** Communication complexity comparison.

Algorithms (Variates)	Entity	PriTKT	[18]
UReg*U*	User	$\left(N+1\right)Z_{p}\left|+1\right|G_{2}|$	$\left(N+3\right)|Z_{p}\left|+3\right|G_{1}|$
UReg*CA*	CA	$1|Z_{p}\left|+2\right|G_{2}|$	$3|Z_{p}\left|+1\right|G_{1}|$
SReg*S*	Seller	$6|Z_{p}\left|+8\right|G_{1}\left|+4\right|G_{2}|$	$2|Z_{p}\left|+2\right|G_{1}|$
SReg*CA*	CA	$2|Z_{p}\left|+1\right|G_{1}|$	$3|Z_{p}\left|+1\right|G_{1}|$
Issue*U*	User	$\left(K+5\right)|Z_{p}\left|+3\right|G_{1}\left|+2\right|G_{2}\left|+1\right|G_{T}|$	$\left(2N+K+8\right)|Z_{p}\left|+\left(2K+5\right)\right|G_{1}|$
Issue*S*	Seller	$9|Z_{p}\left|+2\right|G_{2}|$	$6|Z_{p}\left|+1\right|G_{1}|$
Show*U*	User	$6|Z_{p}\left|+2\right|G_{1}\left|+2\right|G_{2}\left|+1\right|G_{T}|$	$\left(K+10\right)|Z_{p}\left|+9\right|G_{1}|$
Show*V*	Verifier	$3|Z_{p}|$	$2|Z_{p}\left|+4\right|G_{1}|$
DSTrace	Verifier	$|Z_{p}\left|+\right|G_{2}|$	$|G_{1}|$

**Table 6:** Experimental computation costs (ms).

ALGO	Entity	PriTKT	[18]
Setup	CA	44,245	98,124
UKeyGen	User	18.1	1924
SKeyGen	Seller	143.6	1924
UReg*U*	User	642.8	337,149
UReg*CA*	CA	95.5	101,972
SReg*S*	Seller	1024.3	28,551
SReg*CA*	CA	1543.2	5772
${\mathrm{Issue}}_{U}$	User	1971.4	561,049
${\mathrm{Issue}}_{S}$	Seller	908.2	201,506
Show*U*	User	320.4	79,544
Show*V*	Verifier	804.3	69,924
DSTrace	Verifier	18.5	5772

**Table 7:** Experimental storage costs (B).

ALGO	Entity	PriTKT	[18]
pp	CA	348,816	2584
upk	User	272	136
spk	Seller	2112	136
$cred_{u}$	User	256	216
$cred_{s}$	Seller	528	216
tkt	User	704	472
tok	User	1528	1488

**Table 8:** Experimental communication costs (B).

ALGO	Entity	PriTKT	[18]
UReg*U*	User	2352	2568
UReg*CA*	CA	584	256
SReg*S*	Seller	1584	3,52
SReg*CA*	CA	208	256
Issue*U*	User	1856	6640
Issue*S*	Seller	904	376
Show*U*	User	1568	1824
Show*V*	Verifier	120	624
DSTrace	Verifier	312	136

**Table 9:** Computation overhead of Issue and Show (ms).

ALGO	System	Number of Attributess				
		20	40	60	80	100
Issue*U*	PriTKT	1200.5	1723	2242.1	2767.1	3283.5
	[18]	378,196	500,098	622,002	743,903	865,801
Issue*S*	PriTKT	905.6	906.7	908.1	904.5	906.7
	[18]	201,506	201,508	201,505	201,506	201,507
Show*U*	PriTKT	325.9	323.1	324.2	320.2	321.4
	[18]	79,544	79,541	79,545	79,543	79,542
Show*V*	PriTKT	801.1	803.9	802.4	802.3	803.6
	[18]	69,924	69,923	69,925	69,922	69,924

**Table 10:** Communication overheads of Issue and Show (B).

ALGO	System	Number of Attributess				
		20	40	60	80	100
Issue*U*	PriTKT	1856	1856	1856	1856	1856
	[18]	4240	5840	7440	9040	10,640
Issue*S*	PriTKT	904	904	904	904	904
	[18]	376	376	376	376	376
Show*U*	PriTKT	1586	1568	1586	1586	1586
	[18]	1824	1824	1824	1824	1824
Show*V*	PriTKT	120	120	120	120	120
	[18]	624	624	624	624	624

## Data Availability

Data are contained within the article and supplementary materials.

## Supplementary Materials

The following supporting information can be downloaded at: https://www.mdpi.com/article/10.3390/s24020496/s1, Section S1: Correctness Analysis; Section S2: Security Proof; Section S3: Zero-Knowledge Signature of Knowledge; Section S4: Formal Correctness Definition.
