# Supplementary material for: PriTKT: A Blockchain-Enhanced Privacy-Preserving Electronic Ticket System for IoT Devices
Source: Sensors (Basel). 2024 Jan 13;24(2):496. doi: 10.3390/s24020496 (PMC11154337; doi:10.3390/s24020496)
Supplement: Supplementary file 1 [file sensors-24-00496-s001.zip › sensors-2767045-supplementary.pdf]

Supplemental Material

S1. Correctness Analysis

We claim that PriTKT system described in section 4 is correct. Using the properties of bilinear maps, it is trivial to validate that the equations in Setup, SKeyGen and UKeyGen which are used to system initialization and key generation, and Pse. 6 and Pse. 7 which are used to verify the credentials sent to U and S, hold. Similarly, it is straightforward to verify the equations in DSTrace and VerifyDS, which are used to detect double-spending and verify the correctness of double-spending tracing.

We prove that the correctness of the equations in Pse. 8 and Pse. 9 also hold true. The former are used by U and S to issue tickets, while the latter are used by U and V to show tickets.

The following prove that the Issue algorithm is correct. For a valid credential $cred_u = (\widetilde{\sigma}_1, \widetilde{\sigma}_2)$ issued on $\mathbb{A}$ and usk, we have:

$$e(X(Y_0)^{usk} \prod_{i\in[N]} Y_i^{a_i}, \widetilde{\sigma}_1) = e(g, \widetilde{\sigma}_2) \tag{1}$$

which is equivalent to:

$$e((Y_0)^{usk}, \widetilde{\sigma}_1) = e(g, \widetilde{\sigma}_2) e(X \prod_{i\in[N]} Y_i^{a_i}, \widetilde{\sigma}_1)^{-1} \tag{2}$$

Therefore:

$$\begin{aligned}
& e(Y_0^s, \widetilde{\sigma}'_1)(\sigma'_3)^{-1} \\
&= e(Y_0, \widetilde{\sigma}_1^{r\cdot s}) e(Y_0, \widetilde{\sigma}_1^{-r\cdot k}) \\
&= e(Y_0, \widetilde{\sigma}_1^{r(s-k)}) = e((Y_0)^{usk}, \widetilde{\sigma}_1)^{r\cdot c} \\
&= [e(g, \widetilde{\sigma}_2) e(X \prod_{i\in[N]} Y_i^{a_i}, \widetilde{\sigma}_1)^{-1}]^{r\cdot c} \\
&= [e(g, \widetilde{\sigma}'_2) e(g, \widetilde{\sigma}'_1)^{-t} e(X \prod_{i\in[N]} Y_i^{a_i}, \widetilde{\sigma}'_1)^{-1}]^{c} \\
&= [e(g, \widetilde{\sigma}'_2) e(Xg^t \prod_{i\in[N]} Y_i^{a_i}, \widetilde{\sigma}'_1)^{-1}]^{c} \\
&= [e(g, \widetilde{\sigma}'_2) e(X\sigma_1 \prod_{i\in\mathcal{D}} Y_i^{a_i}, \widetilde{\sigma}'_1)^{-1}]^{c}
\end{aligned} \tag{3}$$

and

$$\begin{aligned}
& e(\sigma_1, \prod_{i\in\mathcal{D}\cup\{0\}} \widetilde{Y}_i) \\
&= e(\prod_{i\in[N]\setminus\mathcal{D}} Y_i^{a_i}, \prod_{i\in\mathcal{D}\cup\{0\}} \widetilde{Y}_i) e(g^t, \prod_{i\in\mathcal{D}\cup\{0\}} \widetilde{Y}_i) \\
&= e((\prod_{i\in[N]\setminus\mathcal{D}} Y_i^{a_i}, \widetilde{g})^{\sum_{i\in\mathcal{D}\cup\{0\}} y_i}) e((\prod_{i\in\mathcal{D}\cup\{0\}} Y_i)^t, \widetilde{g}) \\
&= e(\sigma_2, \widetilde{g})
\end{aligned} \tag{4}$$

in addition, we have

$$\begin{aligned}
& [(\sigma'_3)^{-1} \cdot e(Y_0^s, \widetilde{\sigma}'_1)]^{c^{-1}} \\
&= [e(Y_0^k, \widetilde{\sigma}'_1)^{-1} e((Y_0^s, \widetilde{\sigma}'_1)]^{c^{-1}} \\
&= e(Y_0^{s-k}, \widetilde{\sigma}'_1)^{c^{-1}} \\
&= e(Y_0^{c\cdot usk}, \widetilde{\sigma}'_1)^{c^{-1}} \\
&= e(Y_0^{usk}, \widetilde{\sigma}'_1) = \omega
\end{aligned} \tag{5}$$

Eqs. 1, Eqs. 2, Eqs. 3, Eqs. 4 and Eqs. 5 are used to show the correctness of Issue algorithm.

The following prove that the Show algorithm is correct. For a valid ticket $tkt = (\widetilde{T}_1, \widetilde{T}_2)$, we have:

$$e(AB_0^{usk} B_1^{dsid} B_2^{dsrnd} B_3^{VP_{tkt}}, \widetilde{T}_1) = e(g, \widetilde{T}_2) \tag{6}$$

which is equivalent to:

$$e(B_0^{usk}, \widetilde{T}_1) = e(g, \widetilde{T}_2) e(AB_1^{dsid} B_2^{rnd} B_3^{VP_{tkt}}, \widetilde{T}_1)^{-1} \tag{7}$$

Therefore:

$$\begin{aligned}
& e(B_0^{s'}, \widetilde{T}'_1)(T'_3)^{-1} \\
&= e(B_0, \widetilde{T}_1^{r'\cdot s'},) e(B_0, \widetilde{T}_1^{-r'\cdot dsrnd}) \\
&= e(B_0, \widetilde{T}_1^{r'(s'-dsrnd)}) = e(B_0^{usk}, \widetilde{T}_1)^{r'\cdot c'} \\
&= [e(g, \widetilde{T}_2) e(AB_1^{dsid} B_2^{dsrnd} B_3^{VP_{tkt}}, \widetilde{T}_1)^{-1}]^{r'\cdot c'} \\
&= [e(g, \widetilde{T}'_2) e(g, \widetilde{T}'_1)^{-t'} e(AB_1^{dsid} B_2^{dsrnd} B_3^{VP_{tkt}}, \widetilde{T}'_1)^{-1}]^{c'} \\
&= [[e(g, \widetilde{T}'_2) e(Ag^t B_1^{dsid} B_2^{dsrnd} B_3^{VP_{tkt}}, \widetilde{T}'_1)^{-1}]^{c'} \\
&= [e(g, \widetilde{T}'_2) e(AT_1 B_1^{dsid} B_3^{VP_{tkt}}, (\widetilde{T}'_1)^{-1})]^{c'}
\end{aligned} \tag{8}$$

and

$$\begin{aligned}
& e(T_1, \widetilde{B}_0\widetilde{B}_1\widetilde{B}_3) \\
&= e(B_2^{dsrnd}, \widetilde{B}_0\widetilde{B}_1\widetilde{B}_3) e(g^{t'}, \widetilde{B}_0\widetilde{B}_1\widetilde{B}_3) \\
&= e((B_2^{dsrnd})^{b_0+b_1+b_3}, \widetilde{g}) e((B_0B_1B_3)^{t'}, \widetilde{g}) \\
&= e(T_2, \widetilde{g})
\end{aligned} \tag{9}$$

The equations 6, 7, 8 and 9 are utilized to prove the correctness of the Show algorithm.

S2. Security Proof

Let $\Pi_1, \Pi_2, \Pi_3, \Pi_4$ be ZKSoKs, and the formal proofs of the PriTKT system are provided as follows.

S2.1 Unforgeability of Credentials.

***Theorem 1*:** In PriTKT system, the user's credential is unforgeable if the DL assumption holds in $\mathbb{G}_2$ and if URS is unforgeable.

Proof: Suppose an adversary $\mathcal{A}$ aims to breach the credential unforgeability of PriTKT. $\mathcal{A}$ wins the $Exp^{uf_{cred}}$ game when it succeeds in forging a user's attribute credential. Let usk denote the user's private key, and if $\mathcal{A}$ presents the credential, it

proves the knowledge of usk. The adversary's objective is to forge the credential of either a registered or unregistered user in the Issue algorithm. Accordingly, we identify two types of adversaries:

Type-1: $\mathcal{A}$ forges $cred_i$, where $\exists i \in \mathcal{HU}$, and $\mathcal{USK}[i] = usk$;

Type-2: $\mathcal{A}$ forges $cred_i$, where $\forall i \in \mathcal{HU}$, and $\mathcal{USK}[i] \neq usk$;

Lemma 1. If there is a type-1 adversary $\mathcal{A}$ that breaks the unforgeability of credentials with the probability ϵ, then there is a challenger C that breaks the DL assumption with the probability $\frac{\epsilon}{n}$, where n is a bound on the number of honest users.

Proof: Let $(\widetilde{g}, \widetilde{g}^x)$ be a DL challenge in $\mathbb{G}_2$. C use $\widetilde{g}$ as the generator for $\mathbb{G}_2$. C runs $(msk, pp, \mathbb{P}) \leftarrow \mathsf{Setup}(1^\lambda)$, and then runs $\tau \leftarrow \mathsf{SimGen}(1^\lambda)$ to generate a trapdoor of ZKSoK using pp as paramters. C sends $(pp, \mathbb{P})$ to $\mathcal{A}$. When we consider a type-1 forgery, there is an user identity i^* such that $\mathcal{A}$ will try to impersonate the i^*-th honest user in the Issue algorithm. C makes a guess on $i^* \in [n]$ and answers oracles queries as follows:

• $O_{HU}(i)$. If $i \neq i^*$, then C proceeds as usual. Otherwise, it assigns $\mathcal{UPK}[i] = \widetilde{g}^x$ and adds i to $\mathcal{HU}$; it returns $\mathcal{UPK}[i]$.

• $O_{CU}(i, upk)$. If $i \neq i^*$, then C proceeds as usual and aborts otherwise.

• $O_{UReg}(i, \mathbb{A})$. C knows CA's secret msk and so perfectly plays CA's part of this oracle. It can also simulate the honest user i, if $i \neq i^*$. Otherwise, it simulates the ZKSoK of x.

• $O_{HS}(j), O_{SReg}(j), O_{Iss_S}(j, i, \mathcal{D})$. C knows CA and seller's secret and so perfectly simulates these oracles.

• $O_{Iss}(i, j, \mathcal{D})$. C knows the seller's secret ssk and so perfectly plays the seller's part of this oracle. It can also simulate the honest user i, if $i \neq i^*$. Otherwise, it simulates the ZKSoK of x. Note that x only appears in the following contexts: (1) $s = k + c \cdot x$; (2) $\omega = e(Y_0^x, \widetilde{\sigma}'_1)$; (3) Π_3. C computes $\omega = e(Y_0, (\widetilde{g}^x)^{r_u \cdot r})$. Since (1) and (3) use the signature of knowledge, they are easy to simulate.

• $O_{Shw}(i, j)$. If $i \neq i^*$, then C can perfectly play the user's part of this oracle. Otherwise, it runs the algorithm but simulates the ZKSoK of x. Note that x only appears in $s' = dsrnd + c' \cdot x$, it is easy to simulate.

If the guess on i^* is correct, the game is simulated perfectly and the probability of occurrence is $\frac{1}{n}$. In this case, a successful adversary $\mathcal{A}$ proves the ZKSoK of x when it obtains a ticket in Issue algorithm. C can then run the extractor of Π_3 to restore x, which is returned as a valid solution to the DL assumption. The probability of success for C is $\frac{\epsilon}{n}$.

Lemma 2. If there is a type-2 adversary $\mathcal{A}$ that breaks the unforgeability of credentials with the probability ϵ, then there is a challenger C that breaks the unforgeability of the URS scheme with the same probability.

Proof: C runs the unforgeability game of the URS and so receives a public parameters $(\mathbb{G}_1, \mathbb{G}_2, \mathbb{G}_T, g, \widetilde{g}, p, e, X, Y_0, \ldots, Y_N, \widetilde{Y}_0, \ldots, \widetilde{Y}_N, Z_{i,j}, 0 \leqslant i \neq j \leqslant N)$. C runs $\mathsf{Setup}(1^\lambda)$ to genetare remaining parameters $(h, \widetilde{D}, \widetilde{E}_0, \ldots, \widetilde{E}_3, \mathbb{P})$, and then runs $\tau \leftarrow \mathsf{SimGen}(1^\lambda)$ to generate a trapdoor of ZKSoK using pp as paramters. C sends $(pp, \mathbb{P})$ to adversary $\mathcal{A}$. C can ask the oracle of URS, $O_{Sign}(\cdot)$, with an unlimited number of times. C answers oracles queries as follows:

• $O_{HU}(i), O_{HS}(j), O_{CU}(i, upk), O_{Iss}(i, j, \mathcal{D}), O_{Shw}(i, j), O_{Iss_S}(j, i, \mathcal{D})$. C knows users and sellers secret keys and so perfectly simulates these oracles.

• $O_{SReg}(j)$. C knows CA's secret which used to sign the seller's credential and so perfectly simulates this oracle.

• $O_{UReg}(i, \mathbb{A})$. C runs the extractor of Π_2 to recovers the secret key $\mathcal{USK}[i]$ and then submits $(\mathcal{USK}[i], \mathbb{A})$ to the URS signing oracle $O_{Sign}(\cdot)$. It then receives a URS signature $(\sigma_1, \sigma_2, \widetilde{\sigma}_1, \widetilde{\sigma}_2)$ whose first two elements are $1_{\mathbb{G}_1}$. C then discards (σ_1, σ_2) and stores the resulting credential $(\widetilde{\sigma}_1, \widetilde{\sigma}_2)$.

C can handle any oracles queries and never aborts. Therefore, at the end of the game, if $\mathcal{A}$ can prove possession of a credential on disclosed attributes set $\mathbb{D}$ with probability ϵ, then C extracts usk^* from Π_3 and stores the elements $(\sigma_1^*, \sigma_2^*, \widetilde{\sigma}_1'^*, \widetilde{\sigma}_2'^*)$. Since we consider a type-2 forgery, for any honest user i, usk^* must be different from $\mathcal{USK}[i]$, so C constructs a valid derived signature on $(usk^*, \mathbb{D}, \cdot)$.

S2.2 Unforgeability of Tickets.

Theorem 2: In PriTKT system, the user's tickets are unforgeable if the DL assumption holds in $\mathbb{G}_2$ and if URS is unforgeable.

Proof: Let $\mathcal{A}$ be an adversary against the unforgeability of tickets in PriTKT system. When $\mathcal{A}$ wins the game $Exp^{uf_{tkt}}$, $\mathcal{A}$ forges a ticket. Let usk be the user private key, and when $\mathcal{A}$ shows the ticket, the knowledge of usk is proved by $\mathcal{A}$. The adversary wins if they can forge a ticket of an honest user or a ticket of an unregistered user in Show algorithm, therefore we distinguish two types of adversary:

Type-3: $\mathcal{A}$ forges tkt_i, where $\exists i \in \mathcal{HU}$, and $\mathcal{USK}[i] = usk$;

Type-4: $\mathcal{A}$ forges tkt_i, where $\forall i \in \mathcal{HU}$, and $\mathcal{USK}[i] \neq usk$;

Lemma 3. If there is a type-3 adversary $\mathcal{A}$ that breaks the unforgeability of tickets with the probability ϵ, then there is a challenger C that breaks the DL assumption with the probability $\frac{\epsilon}{n}$, where n is a bound on the number of honest users.

Proof: Assumptions and oracles definitions are consistent with ***lemma 1***. In this case, a successful adversary $\mathcal{A}$ proves knowledge of x when it shows the ticket in Show algorithm. C can then run the extractor of ZKSoK to restore x, which is returned as a valid solution to the DL assumption. The probability of success for C is $\frac{\epsilon}{n}$.

Lemma 4. If there is a type-4 adversary $\mathcal{A}$ that breaks the unforgeability of tickets with the probability ϵ, then there is a challenger C that breaks the unforgeability of the URS scheme with the probability $\frac{\epsilon}{m}$, where m is a bound on the number of honest sellers.

Proof: C runs the unforgeability game of the URS scheme and so receives a public key $spk^* = (A^*, B_0^*, \ldots, B_3^*, \widetilde{B}_0^*, \ldots, \widetilde{B}_3^*, C_{0,2}^*, C_{1,2}^*, C_{3,2}^*)$ and bilinear paring paramaters $pp = (\mathbb{G}_1, \mathbb{G}_2, \mathbb{G}_T, g, \widetilde{g}, p, e)$. C runs $\mathsf{Setup}(1^\lambda)$ to generate remaining parameters using pp as paramters, and then runs $\tau \leftarrow \mathsf{SimGen}(1^\lambda)$ to generate a trapdoor of ZKSoK. C sends $(pp, \mathbb{P})$ to $\mathcal{A}$. C can ask

the oracle of URS, $O_{Sign}(\cdot)$, with an unlimited number of times. Since we consider a type-4 forgery, there is an identity j^* such that $\mathcal{A}$ will try to forge a ticket that can be verified by the j^*-th honest seller's publick key. C then makes a guess on $j^* \in [m]$. C can then answers oracle queries as follows.

• $O_{HU}(i), O_{CU}(i, upk), O_{UReg}(i, \mathbb{A}), O_{Shw}(i, j)$. C knows CA and user's secret key and so perfectly simulates these oracles.

• $O_{HS}(j)$. If $j \neq j^*$, then C proceeds as usual. Otherwise, it assign $\mathcal{SPK}[j] = spk^*$ and adds j to $\mathcal{HS}$; it returns $\mathcal{SPK}[j]$.

• $O_{SReg}(j)$. If $j \neq j^*$, then C proceeds as usual. Otherwise, it simulates the signature of knowledge of $\mathcal{SSK}[j]$.

• $O_{Iss_S}(j, i, \mathcal{D})$. If $j \neq j^*$, then C proceeds as usual. Otherwise, C simulates the signature of knowledge of $\mathcal{SSK}[j]$, and then runs the extractor of Π_3 to recovers the secret ($\mathcal{USK}[i]$, $dsid'$, $dsrnd$) and then submits ($\mathcal{USK}[i], dsid' + dsid'', dsrnd$, VP_{tkt}) to the signing oracle $O_{Sign}(\cdot)$. It then receives a URS signature $(T_1^*, T_2^*, \widetilde{T}_1^*, \widetilde{T}_2^*)$ whose first two elements are $1_{\mathbb{G}_1}$. C then discards (T_1^*, T_2^*) and stores the resulting ticket $(\widetilde{T}_1^*, \widetilde{T}_2^*)$.

• $O_{Iss}(i, j, \mathcal{D})$. C knows the user's secret key and so perfectly simulates the user's side of this oracle. If $j \neq j^*$, then C proceeds as usual. Otherwise, it performs the same operation as $O_{Iss_S}(j, i, \mathcal{D})$.

At the end of the game, $\mathcal{A}$ can prove possession of a ticket issued by seller j with probability ϵ. If $j \neq j^*$, then it aborts and the probability of termination is $\frac{m-1}{m}$. Otherwise, C extracts usk^* from the ZKSoK and stores the elements $(T_1^*, T_2^*, \widetilde{T}_1'^*, \widetilde{T}_2'^*)$. Since we consider a type-4 forgery, for any honest user i, usk^* must be different from $\mathcal{USK}[i]$, so C constructs a valid derived signature on $(usk^*, dsid, dsrnd, VP_{tkt})$ with probability $\frac{\epsilon}{m}$.

S2.3 Unlinkability of Credentials.

Theorem 3: In PriTKT system, the user's credential is unlinkable if the DDH assumption holds in $\mathbb{G}_2$.

Proof: If there is an adversary $\mathcal{A}$ that breaks the unlinkability of credentials with the probability ϵ in Issue algorithm, then there is a challenger C that breaks the DDH assumption in $\mathbb{G}_2$ with the probability $\frac{\epsilon}{n}$, where n is a bound on the number of honest users.

Let $(\widetilde{g}, \widetilde{g}^x, \widetilde{g}^y, \widetilde{g}^z)$ be a DDH challenge in $\mathbb{G}_2$, C runs Setup $(1^\lambda) \rightarrow (msk, pp, \mathbb{P})$ by using $\widetilde{g}$ as the generator for $\mathbb{G}_2$, and then runs $\tau \leftarrow$ SimGen(1^λ) to generate a trapdoor of ZKSoK using pp as paramters. C sends $(pp, \mathbb{P})$ to $\mathcal{A}$. C guesses the identity of the user i_b, the user will have the credential $cred_{i^*}$ targeted by $\mathcal{A}$. C answers the first stage of the oracles query as follows .

• $O_{HS}(j), O_{CS}(j), O_{SReg}(j)$. C knows CA and seller's secret keys and so perfectly simulates these oracles.

• $O_{HU}(i)$. If $i \neq i^*$, then C proceeds as usual. Otherwise, it assigns $\mathcal{UPK}[i] = \widetilde{g}^x$, and adds i to $\mathcal{HU}$; it returns $\mathcal{UPK}[i]$.

• $O_{UReg}(i, \mathbb{A})$. C knows CA's secret msk and so perfectly simulates CA's side of this oracle. It can also simulate the honest user i, if $i \neq i^*$. Otherwise, it simulates the ZKSoK of x.

• $O_{Iss_U}(i, j, \mathcal{D})$. C can simulate the honest user i, if $i \neq i^*$. Otherwise, it simulates the ZKSoK of x. Note that x only appears in the following contexts: (1) $s = k + c \cdot x$; (2) $\omega = e(Y_0^x, \widetilde{\sigma}_1')$; (3) Π_3. C compute $\omega = e(Y_0, (\widetilde{g}^x)^{r_u \cdot r})$. Since (1) and (3) use the ZKSoK, it is easy to simulate given that he has already computed ω.

• $O_{Iss}(i, j, \mathcal{D})$. C knows S's secret ssk and so perfectly plays the S's side of this oracle. If $i \neq i^*$, then C proceeds as usual. Otherwise, C performs the same operation as $O_{Iss_U}(i, j, \mathcal{D})$.

• $O_{Shw}(i, j)$. C can simulate any honest user i, if $i \neq i^*$. Otherwise, it simulates the ZKSoK of x.

At some point in the game, $\mathcal{A}$ outputs the indices i_0 and i_1 of two credentials along with a set of attributes $\mathbb{D}^*$ and an honest seller j^*. If $i_b \neq i^*$, then C aborts. Otherwise, it proceeds as follows.

• $O_{Iss_U}(i_b, j^*, \mathcal{D}^*)$. C first selects a random α and computes:

$$\sigma_1^* = g^\alpha$$
$$\sigma_2^* = (\sigma_1^*)^{\sum_{i \in \mathcal{D}^* \cup \{0\}} y_i}$$
$$\widetilde{\sigma}_1'^* = \widetilde{g}^y$$
$$\widetilde{\sigma}_2'^* = (\widetilde{g}^z)^{y_0} (\widetilde{\sigma}'^*)^{x + \sum_{i \in \mathbb{A}} y_i \cdot a_i}$$

The simulation of the ZKSoK of x is consistent with that of $O_{Iss_U}(\cdot)$.

• $O_{Shw}(i^*, j^*)$. C simulates the ZKSoK of x.

If $x \cdot y = z$, then, by setting $t = \alpha - \sum_{i \in [N] \setminus \mathcal{D}^*} y_i \cdot a_i$, we can see that $(\sigma_1^*, \sigma_2^*, \widetilde{\sigma}_1'^*, \widetilde{\sigma}_2'^*)$ are distributed as in the Issue algorithm. Otherwise, z is random, which means that $\widetilde{\sigma}_2'^*$ is random. Since $(\sigma_1^*, \sigma_2^*, \widetilde{\sigma}_1'^*, \widetilde{\sigma}_2'^*)$ are independent of x and $\mathbb{D}^*$, $\mathcal{A}$ cannot succeed in this game with non negligible advantage ϵ. Therefore, the behaviour of $\mathcal{A}$ can be used to solve the DDH assumption in $\mathbb{G}_2$, unless C aborts. The advantage of C is at least $\frac{\epsilon}{n}$.

S2.4 Unlinkability of Tickets.

Theorem 4: In PriTKT system, the user's tickets are unlinkable if the DDH assumption holds in $\mathbb{G}_2$.

Proof: If there is an adversary $\mathcal{A}$ that breaks the unlinkability with the probability ϵ in Show algorithm, then there is a challenger C that breaks the DDH assumption in $\mathbb{G}_1$ with the probability $\frac{\epsilon}{n}$, where n is a bound on the number of honest users.

The game setup for ***Theorem 4*** is the same as for ***Theorem 3*** except for the following oracles.

• $O_{Iss_U}(i^*, j^*, \mathcal{D}^*)$. The simulation is the same as for $O_{Iss_U}(i, j, \mathcal{D})$ in ***Theorem 3***.

• $O_{Shw}(i^*, j^*)$. C selects a random α and computes:

$$T_1^* = g^\alpha$$
$$T_2^* = (T_1^*)^{b_0 + b_1 + b_3}$$
$$\widetilde{T}_1'^* = \widetilde{g}^y$$
$$\widetilde{T}_2'^* = (\widetilde{g}^z)^{b_0} (\widetilde{T}_1'^*)^{a + b_1 \cdot dsid + b_2 \cdot dsrnd + b_3 \cdot VP_{tkt}}$$

and then C simulate the signature of knowledge of x.

If $x \cdot y = z$, then, by setting $t = \alpha - (b_2 \cdot dsrnd)$, we see that $(T_1^*, T_2^*, \widetilde{T}_1'^*, \widetilde{T}_2'^*)$ are distributed as in the Show algorithm. Otherwise, z is random, which means that $\widetilde{T}_2'^*$ is random. Since $(T_1^*, T_2^*, \widetilde{T}_1'^*, \widetilde{T}_2'^*)$ are independent of x, $\mathcal{A}$ cannot succeed in this game with non negligible advantage ϵ. Therefore, the behaviour of $\mathcal{A}$ can be used to solve the DDH assumption in $\mathbb{G}_2$, unless C aborts. The advantage of C is then at least $\frac{\epsilon}{n}$.

S2.5 Framing-resistance.

***Theorem 5*:** PriTKT system is framing-resistance if the DL assumption holds in $\mathbb{G}_2$.

Proof: If there is an adversary $\mathcal{A}$ that breaks the framing-resistance with the probability ϵ, then there is a challenger C that breaks the DL assumption in $\mathbb{G}_2$ with the probability $\frac{\epsilon}{n}$, where n is a bound on the number of honest users.

The setup and simulations of oracles are the same as that of ***Lemma 1***. C perfectly simulates $Exp^{fr}(\mathcal{A}, \lambda)$. At some point, $\mathcal{A}$ outputs $(dsblame, upk')$ with non negligible advantage ϵ. If $upk' \neq \mathcal{UPK}[i^*]$, then C aborts. Otherwise, C outputs $x = dsblame$. C outputs the correct discrete logarithm x, if $upk' = \mathcal{UPK}[i^*] = \widetilde{g}^x$. The advantage of C is then at least $\frac{\epsilon}{n}$.

S3. Zero-Knowledge Signature of Knowledge

The zero-knowledge signature of knowledge mentioned in section 5.2 can be instantiated by Fiat-Shamir paradigm [51].

S3.1 Details of Π_1 *and* Π_1'.

The proof $\Pi_1 = \Pi_1' = \mathsf{ZKSoK}\{(a, b_0, b_1, \ldots, b_3) : A = g^a \wedge B_i = g^{b_i}, 0 \leqslant i \leqslant 3\}$ is computed as below.

(1) S select $(\beta_0, \ldots \beta_4) \xleftarrow{R} \mathbb{Z}_p^5$ and computes $P_0 = g^{\beta_0}, \cdots, P_4 = g^{\beta_4}$, $c_1 = H(A||B_0||\ldots||B_3||P_0||\ldots||\ P_4)$ and $t_0 = \beta_0 - a \cdot c_1$, $t_1 = \beta_1 - b_0 \cdot c_1, \ldots, t_4 = \beta_4 - b_3 \cdot c_2$. S send $\Pi_1 = (c_1, t_0, \ldots, t_4)$ to CA.

(2) CA verifies $c_1 \stackrel{?}{=} H(A||B_0||\ldots||B_3||g^{t_0}A^{c_1}||\ g^{t_1}B_0^{c_1}||\ldots||g^{t_4}B_4^{c_1})$.

S3.2 Details of Π_2.

The proof $\Pi_2 = \mathsf{ZKSoK}\{usk : upk = \widetilde{g}^{usk}\}$ is computed as below.

(1) U select $\alpha_1 \xleftarrow{R} \mathbb{Z}_p$ and compute $R_1 = \widetilde{g}^{\alpha_1}$, $c_2 = H(upk||R_1)$, $s_1 = \alpha_1 - usk \cdot c_2$. U sends $\Pi_2 = (c_1, s_1)$ to CA.

(2) CA verifies $c_2 \stackrel{?}{=} H(upk||\widetilde{g}^{s_1}upk^{c_2})$.

S3.3 Details of Π_3.

The proof $\Pi_3 = \mathsf{ZKSoK}\{(usk, dsid', dsrnd) : \omega = e(Y_0^{usk}, \widetilde{\sigma}_1') \wedge psu = \widetilde{B}_0^{usk}\widetilde{B}_1^{dsid'}\widetilde{B}_2^{dsrnd}\}(nonce)$ is computed as below.

(1) U select $(\alpha_u, \alpha_d, \alpha_r) \xleftarrow{R} \mathbb{Z}_p^3$ and computes $\widehat{R_d} = e(Y_0^{\alpha_u}, \widetilde{\sigma}_1')$, $\widetilde{R_p} = \widetilde{B}_0^{\alpha_u}\widetilde{B}_2^{\alpha_d}\widetilde{B}_2^{\alpha_r}$, $c_3 = H(\omega||psu||\widehat{R_d}||\ \widetilde{R_p}||nonce)$ and $s_u = \alpha_u - usk \cdot c_3, s_d = \alpha_d - dsid' \cdot c_3, s_r = \alpha_r - dsrnd \cdot c_3$. U sends $\Pi_3 = (c_3, s_u, s_d, s_r)$ to S.

(2) S verifies $c_3 \stackrel{?}{=} H(\omega||psu||e(Y_0^{s_u}, \widetilde{\sigma}_1')\omega^{c_3})||\widetilde{B}_0^{s_u}\widetilde{B}_1^{s_d}\widetilde{B}_2^{s_r}\cdot psu^{c_3}||nonce)$.

S3.4 Details of Π_4.

The proof $\Pi_4 = \mathsf{ZKSoK}\{(t', dsrnd) : T_1 = g^{t'}B_2^{dsrnd} \wedge T_3' = e(B_0^{dsrnd}, \widetilde{T}_1')\}(nonce)$ is computed as below.

(1) U select $(\alpha_t, \alpha_r) \xleftarrow{R} \mathbb{Z}_p^2$ and computes $R_1 = g^{\alpha_t}B_2^{\alpha_r}$, $\widehat{R_2} = e(B_0^{\alpha_r}, \widetilde{T}_1')$, $c_4 = H(T_1||T_3'||R_1||\widehat{R_2}||nonce)$ and $s_t = \alpha_t - t' \cdot c_4$, $s_r = \alpha_r - dsrnd \cdot c_4$. U sends (c_4, s_t, s_r) to V.

(2) V verifies $c_3 \stackrel{?}{=} H(T_1||T_3'||g^{s_t}B_2^{s_r}T_1^{c_4}||e(B_0^{s_r}, \widetilde{T}_1')\cdot(T_3')^{c_4}||nonce)$.

S4. Formal Correctness Definition

(1) The tickets produced by the Issue algorithm must be verifiable by the Show;

$$Pr\left[\begin{array}{c|l} \mathsf{Show}(\mathsf{U}(usk, tkt, VP_{tkt}, spk, pp) \leftrightarrow \mathsf{V}(spk, pp) \to ((dsid, dstrace), 1)) & \mathsf{Setup}(1^\lambda) \to (msk, pp, \mathbb{P}); \\ & \mathsf{SKeyGen}(pp) \to (ssk, spk); \\ & \mathsf{SReg}(\mathsf{S}(ssk, spk, pp) \leftrightarrow \mathsf{CA}(msk, spk, pp)) \to cred_s; \\ & \mathsf{UKeyGen}(pp) \to (usk, upk); \\ & \mathsf{UReg}(\mathsf{U}(usk, upk, \mathbb{A}, pp) \leftrightarrow \mathsf{CA}(msk, upk, pp)) \to cred_u; \\ & \mathsf{Issue}(\mathsf{U}(usk, cred_u, \mathcal{D}, \mathbb{A}, pp) \leftrightarrow \mathsf{S}(ssk, cred_s, pp, \mathbb{P})) \to ((tkt, VP_{tkt}), 1) \end{array}\right] = 1$$

(2) The $\mathsf{DSTrace}$ algorithm must be able to track users who attempt double-spending behavior

$$Pr\left[\begin{array}{c|l} \mathsf{DSTrace}(dsid, dstrace, \overline{dsid}, \overline{dstrace}, spk, pp) \to (dsblame, upk'), \mathsf{VerifyDS}(dsblame, upk') \to 1 & \mathsf{Setup}(1^\lambda) \to (msk, pp, \mathbb{P}); \\ & \mathsf{SKeyGen}(pp) \to (ssk, spk); \\ & \mathsf{SReg}(\mathsf{S}(ssk, spk, pp) \leftrightarrow \mathsf{CA}(msk, spk, pp)) \to cred_s; \\ & \mathsf{UKeyGen}(pp) \to (usk, upk); \\ & \mathsf{UReg}(\mathsf{U}(usk, upk, \mathbb{A}, pp) \leftrightarrow \mathsf{CA}(msk, upk, pp)) \to cred_u; \\ & \mathsf{Issue}(\mathsf{U}(usk, cred_u, \mathcal{D}, \mathbb{A}, pp) \leftrightarrow \mathsf{S}(ssk, cred_s, pp, \mathbb{P})) \to ((tkt, VP_{tkt}), 1) \\ & \mathsf{Show}(\mathsf{U}(usk, tkt, VP_{tkt}, spk, pp) \leftrightarrow \mathsf{V}(spk, pp) \to ((dsid, dstrace), 1)); \\ & \mathsf{Show}(\mathsf{U}(usk, tkt, VP_{tkt}, spk, pp) \leftrightarrow \mathsf{V}(spk, pp) \to ((\overline{dsid}, \overline{dstrace}), 1)); \end{array}\right] = 1$$
